# Progressive dry-core-wet-rim hydration trend in a nested-ring topology of protein binding interfaces

**DOI:** 10.1186/1471-2105-13-51

**Published:** 2012-03-27

**Authors:** Zhenhua Li, Ying He, Limsoon Wong, Jinyan Li

**Affiliations:** 1 Bioinformatics Research Center at the School of Computer Engineering, Nanyang Technological University, Singapore 639798, Singapore; 2 School of Computing, National University of Singapore, Singapore 117417, Singapore; 3 Advanced Analytics Institute, University of Technology Sydney, Sydney, Australia

## Abstract

**Background:**

Water is an integral part of protein complexes. It shapes protein binding sites by filling cavities and it bridges local contacts by hydrogen bonds. However, water molecules are usually not included in protein interface models in the past, and few distribution profiles of water molecules in protein binding interfaces are known.

**Results:**

In this work, we use a tripartite protein-water-protein interface model and a nested-ring atom re-organization method to detect hydration trends and patterns from an interface data set which involves immobilized interfacial water molecules. This data set consists of 206 obligate interfaces, 160 non-obligate interfaces, and 522 crystal packing contacts. The two types of biological interfaces are found to be drier than the crystal packing interfaces in our data, agreeable to a hydration pattern reported earlier although the previous definition of immobilized water is pure distance-based. The biological interfaces in our data set are also found to be subject to stronger water exclusion in their formation. To study the overall hydration trend in protein binding interfaces, atoms at the same burial level in each tripartite protein-water-protein interface are organized into a ring. The rings of an interface are then ordered with the core atoms placed at the middle of the structure to form a nested-ring topology. We find that water molecules on the rings of an interface are generally configured in a dry-core-wet-rim pattern with a progressive level-wise solvation towards to the rim of the interface. This solvation trend becomes even sharper when counterexamples are separated.

**Conclusions:**

Immobilized water molecules are regularly organized in protein binding interfaces and they should be carefully considered in the studies of protein hydration mechanisms.

## Background

Water is an important component of biomolecules that is crucial to their formation and association [1], particularly in proteins folding [2] and binding [3]. Many studies have been carried out, by energetic model/experiment or statistical analysis, to uncover the precise roles of water in protein-protein binding. It is widely understood that water molecules can shape the binding sites by filling cavities and can bridge local contacts by hydrogen bonds [4,5]. Although its importance has long been recognized, water is usually excluded in protein binding interface modeling. An interface is often defined according to the change of the solvent accessibility of the residues before and after the binding [6,7], or by the distance between the two chains in the complex [8,9]. As these definitions do not involve water molecules, those residues that are in contact with the other chain indirectly through water molecules--e.g., wet spot residues [10,11]--are missing in these interface models. The size of an interface is therefore underestimated. Actually, wet spots can account as much as 14.5% of the interface residues [10]. As the missing residues are more likely to be in the interface than at the surface in terms of their mobility and energy contribution [10,11], it is unreasonable to overlook interfacial water molecules even when the study is only focused on interfacial residues. Water molecules have also been ignored in most protein-protein interaction studies, especially those in computational approaches. For example, water is rarely considered in protein docking [12], interface analysis [6,13,14], interface classification [15-18], etc.

Few results are reported about the spatial arrangement of water molecules and their solvation trend in protein binding interfaces. An earlier work [19] pioneered the study of hydration patterns in protein interfaces, however, their patterns are isolated only within individual interfaces, which were not derived as a general trend. Their definition of interfacial water is prone of including many exposed water molecules. As some of their interfacial water molecules are actually not in interfaces at all, bias may be introduced to the analysis when the study steps to the fine solvation trend in protein interfaces.

Recently, we introduced a tripartite model of protein binding interfaces [20]. Under this model, an interface is defined as an object of three compartments: the two binding sites of the two interacting chains and the interfacial water molecules. The interfacial water molecules are determined by a recursive computational method. As this newly proposed protein binding interface model is different from traditional definitions of protein binding interface, we named it a *protein-water-protein *interface, or a tripartite interface. A protein-water-protein interface can be represented by a tripartite graph, in which the nodes represent the residues or atoms, depending on the level of the study, and the edges are the contacts among them.

In this work, we conduct a topological analysis of water molecules in protein-water-protein interfaces. The distribution profiles of water molecules in three types of interfaces: obligate interfaces, non-obligate interfaces, and crystal packing contacts are investigated. In the analysis, a feature of atoms and residues, called *burial level*, is sophisticatedly explored. Burial level is defined with respect to an atomic contact network of a protein complex, describing the extent an atom or residue is buried in the protein complex. The atoms of an interface are then organized as a nested-ring topology where atoms at the same burial level in the interface are grouped into level-wise rings. We examine both overall and level-wise views of water arrangements in the interface and on the rings. We find that the interior of protein binding interfaces is not homogeneously the same everywhere in terms of a variety of properties such as wetness, water detectablity, polarity and mobility. Moreover, water molecules in protein binding interfaces are distributed in a dry-core-wet-rim style, suggesting that the solvation of protein interfaces occurs progressively ring-by-ring from core to rim in protein binding interfaces. It is also found that the function of an interaction seems to be another constraint of the associated water arrangement. All of these results indicate that water is an active player in protein binding interfaces and should be considered in the studies of protein binding interfaces.

## Results

### Detectability of water molecules at different burial levels of protein interfaces

The amount of water molecules (in a protein complex) that can be detected by X-ray crystallography is closely correlated with the resolution at which the crystal structure is solved [21]. A previous work also found that the quality of interfacial water information is subject to the resolution of the crystal structure [19]. We investigated correlations between the wetness and resolution of crystal structures of protein interfaces. The average correlation coefficients between the wetness of an interface and the resolution (the resolution value) of the crystal structures of the obligate, non-obligate and crystal packing interfaces in our data are negative, being -0.4015, -0.5460 and -0.5632 respectively. This indicates that water-related properties of protein interface depend on the detectability of the water molecules. This observation is consistent with previous results reported by Rodier *et al. *[19].

**Figure 1:**
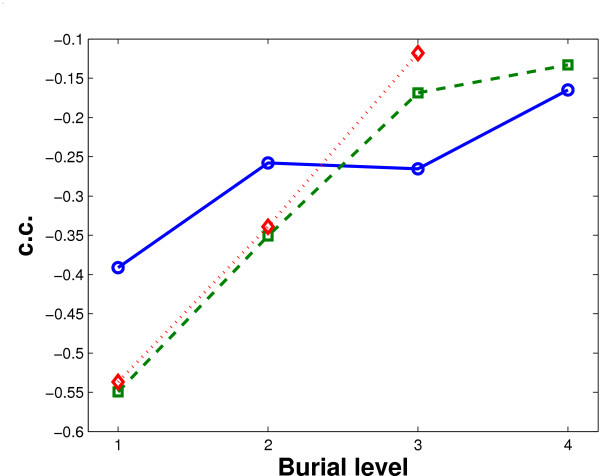
**Relation between water information quality and burial level**. The figure shows correlation coefficients (c.c.) between level-wise wetness and crystal structure resolution at different burial levels in obligate (solid blue, circle marker), non-obligate (dashed green, square marker) and crystal packing (dotted red, diamond marker) interfaces.

We are especially interested in the quality of water information at the core of protein binding interfaces by comparing the quality of water information at different burial levels. We find that the amount of deeply buried water molecules is less correlated with the crystal structure resolutions. That is, as the burial level goes deeper, the correlation becomes weaker; see Figure 1. Thus water molecules in a protein or protein complex cannot be classified simply as exposed or buried. Rather, their properties change gradually when they step into the center of the interface away from the bulk solvent. On the whole, the amount of water molecules is under reported as roughly reported by [21,22]. More importantly, the observation here implies that water molecules at the core of an interface are closer to the completeness (the real amount of water molecules) than those at the other parts. This has promoted our confidence on the quality of our results on the buried water molecules in the core part.

### Wetness of different types of interfaces

Table 1 shows wetness-related statistics of the obligate interfaces, non-obligate interfaces, and crystal packing contacts in our data set. The significance of the differences in wetness, average polarity and relative water burial level are tested by the one-sided Mann-Whitney U test [23] between the obligate and non-obligate interfaces and between the biological and crystal packing interfaces. The p-values are shown in Table 2. In general, the difference between the biological interfaces and crystal packing interfaces is more pronounced than that between the obligate and non-obligate interfaces, both of which are biological interfaces.

**Table 1:** Summary of water related properties of interfaces.

Interaction type	**OB**^***a***^	**NO**^***b***^	**BIO**^***c***^	**CP**^***d***^	All
No. of interfaces	206	160	366	522	888
avg. No. of atoms	610.6 ± 354.2	318.0 ± 131.7	482.7 ± 314.9	186.0 ± 91.8	308.3 ± 259.1
avg. No. of water	28.68 ± 24.03	12.95 ± 9.72	21.80 ± 20.65	10.13 ± 8.46	14.94 ± 15.83
avg. wetness	0.044 ± 0.023	0.039 ± 0.021	0.042 ± 0.022	0.053 ± 0.031	0.049 ± 0.029
avg. WBL	1.577 ± 0.391	1.414 ± 0.281	1.506 ± 0.356	1.282 ± 0.267	1.374 ± 0.326
avg. polarity	0.366 ± 0.026	0.385 ± 0.027	0.374 ± 0.028	0.398 ± 0.038	0.388 ± 0.036
avg. rWBL	1.052 ± 0.172	1.084 ± 0.169	1.066 ± 0.171	1.134 ± 0.196	1.106 ± 0.189
avg. planarity	4.815 ± 1.547	3.960 ± 0.777	4.442 ± 1.337	3.354 ± 0.557	3.803 ± 1.098

Summary information of interfacial water molecules at three different types of interfaces. ^*a*^: obligate interfaces, ^*b*^: non-obligate interfaces, ^*c*^: crystal packing interfaces

**Table 2:** Difference between types of interfaces.

Property	Wetness	Polarity	rWBL
OB vs. NO	0.0260	4.2730 × 10^-10^	0.0541
BIO vs. CP	2.3446 × 10^-7^	2.4387 × 10^-25^	2.6622 × 10^-5^

Significance of the difference between different types of interfaces by the one-sided Mann-Whitney U test

**Figure 2:**
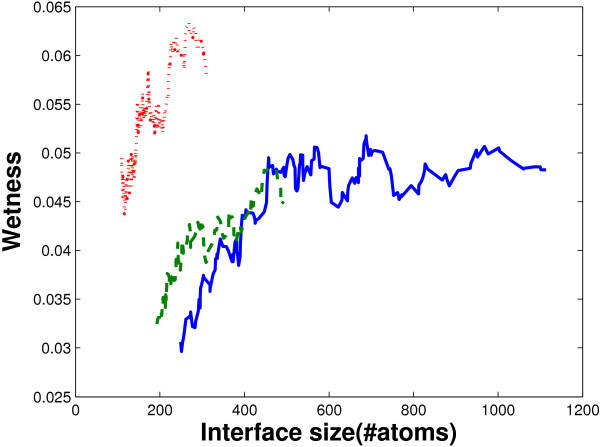
**Relation between interface size and wetness**. The plot is generated by calculating the average wetness of the 41 interfaces with the closest interface size in obligate (solid blue), non-obligate (dashed green) and crystal packing (dotted red) interfaces.

The obligate interfaces are of the largest size, and are capable of holding more water molecules. More specifically, there are about 29 water molecules per interface in the obligate interactions, far more than that in the non-obligate interactions (13 per interface). The crystal packing interfaces are significantly smaller than the non-obligate interfaces; however, they possess almost the same number of water molecules (10 per interface) as the non-obligate interfaces. It has been reported that the number of water molecules held by an interface is correlated with the size of the interface [19]. This correlation is also observed in our data. The correlation coefficients between the number of water molecules and the number of atoms in an interface are 0.8232, 0.6177 and 0.6540 for the obligate, non-obligate and crystal packing interfaces, respectively. Moreover, the wetness of an interface is also bounded by its size. In Figure 2, the relationship between the wetness and interface size is shown. It can be noted that, when interface size is small (less than 500 atoms), wetness is strictly bounded by interface size for both the obligate and non-obligate interfaces. On the other hand, in the crystal packing interfaces, although it seems that the average wetness is somehow related to interface size, but the wetness values are extremely high. The average wetness of the crystal packing interfaces with less than 200 atoms is as high as 0.050, a very high value for such small interfaces. Note that, this correlation between interface size and wetness is due to the upper bound of the wetness of an interface of a certain size. The interface can be very dry for interface of any size. A possible reason why the wetness is bounded by interface size is that, to immobilize a water molecule into an interface, multiple interacting atoms in the interface are required. Then, interfaces of a larger size can offer more water-holding atom clusters, resulting in wetter interfaces.

**Figure 3:**
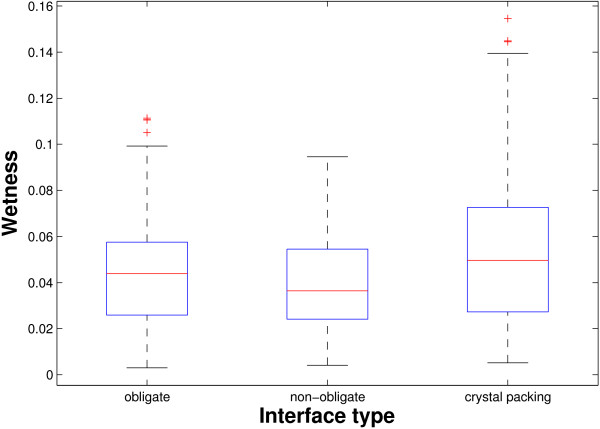
**Relation between wetness and interface type**. The figure shows the box plots of wetness in obligate, non-obligate and crystal packing interfaces. The red line in the central of a box is the median; the upper and lower edges of a box are the 75% (*v*_75_) and 25% (*v*_25_) percentiles, respectively; and the two outer bars indicate the most extreme data points. All boxes are drawn without considering the outliers (red dots). A value is a outlier if it is larger than *v*_75 _+ 1.5(*v*_75 _- *v*_25_) or smaller than *v*_25 _- 1.5(*v*_75 _- *v*_25_).

Figure 3 shows the wetness distributions of the three types of interfaces. Combining with column 2 of Table 2, it can be observed that the obligate interfaces tend to be wetter than the non-obligate interfaces; and these biological interfaces are drier than the crystal packing interfaces. Generally, obligate interactions possess large binding affinity. The binding is so strong that the interaction partners have to be denatured to be separated from each other. The high wetness of the obligate interfaces (compared to the non-obligate interfaces in our data) and the even higher wetness of the crystal packing interfaces (compared to the obligate interfaces) suggest that there is no simple correlation between amount of water and the binding strength.

### Level-wise distribution of water in protein interfaces

Given a tripartite interface, we partition its atoms according to their burial levels. Atoms at the same burial level are organized as a ring. The ring of "core atoms" consists of those atoms with the highest burial level in the interface. The rings are then ordered with the ring of core atoms in the middle. Thus, a tripartite interface can be viewed as a nested-ring structure. The ring of core atoms is denoted by *O*_0_, the ring closest to the core is denoted by *O*_1_, similarly for *O*_2_, etc. We examine how water molecules are distributed in these rings of an interface by looking at level-wise wetness. As the highest burial level varies a lot from one interface to another, we choose the core of interfaces as the starting point to see the change trend of level-wise wetness towards to the rim of the interfaces.

From Table 3, we can see that a progressive dry-core-wet-rim water distribution pattern exists in protein interfaces, with the core *O*_0 _more desolvated than the other rings that are closer to the rim. Similarly to the proportion of water molecules (i.e., wetness), the proportion of polar atoms (i.e., polarity) also increases when the burial level goes from core to rim, even in the crystal packing interfaces. Thus, although the overall wetness and polarity of the three types of interfaces are different, the change trend of their level-wise wetness and polarity is the same from core to rim, following a cone pattern.

**Table 3:** Level-wise property of interfaces.

Burial level	#water	level wetness	level polarity
*O*_0 _(core)	1.048/0.916/1.011	0.032/0.029/0.034	0.315/0.314/0.336
*O*_1_	4.733/3.488/4.510	0.044/0.040/0.061	0.329/0.354/0.368
*O*_2_	10.18/6.959/8.107	0.051/0.050/0.084	0.355/0.384/0.402
*O*_3_	17.15/9.522/13.95	0.068/0.063/0.114	0.377/0.381/0.388
*O*_4_	23.26/-/-	0.073/-/-	0.375/-/-

Wetness and polarity at different burial levels. The three values in each cell correspond to obligate, non-obligate and crystal packing interfaces, respectively

**Figure 4:**
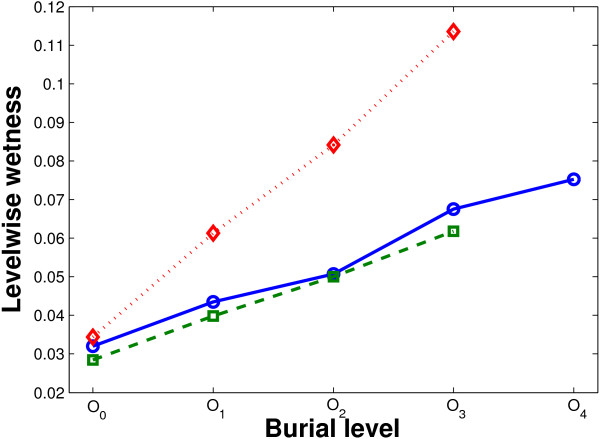
**Level-wise wetness at different burial levels**. Smooth increase of wetness is observed in obligate (solid blue, circle marker), non-obligate (dashed green, square marker) and crystal packing (dotted red, diamond marker) interfaces. The average *Δwetness *per level is 0.011, 0.011 and 0.026 for obligate, non-obligate and crystal packing interfaces.

For more visual clarity of the change trend of level-wise wetness, three curves corresponding to the three types of interfaces are plotted as shown in Figure 4. A clear smooth increase in wetness from core to rim is observed in the obligate, non-obligate, as well as crystal packing interfaces.

**Figure 5:**
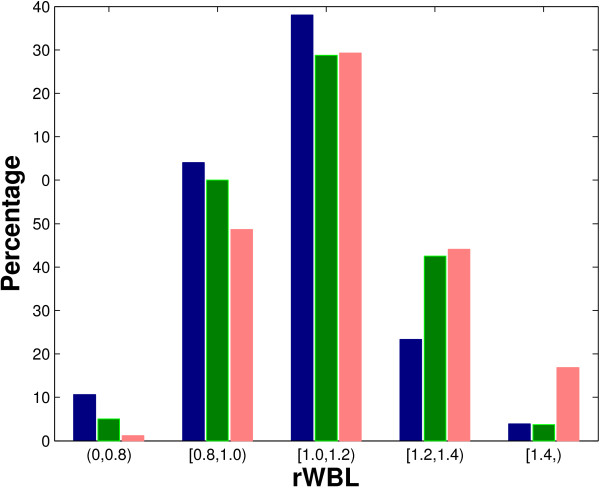
**Comparing the rWBL of the three types of interfaces**. The figure shows the distribution of rWBL of obligate (dark blue), non-obligate (green) and crystal packing (light red) interfaces.

The crystal packing interfaces have the largest inter-level wetness differences. However, this does not indicate that crystal packing interfaces are most capable of excluding interfacial water from core to rim. Rather, this is due to the small size of crystal packing interfaces and the extremely high wetness of their outer rims. To quantitatively understand the extent to which water molecules are "excluded" from the core of an interface, we introduce the relative water burial level (rWBL, see Methods) as the average burial level of water molecules in the interface divided by the average burial level of all the interfacial atoms. If the rWBL of an interface is high, its water molecules are deeply buried in the interface; if it is low, the water molecules are distributed in the rim of the interface. The distribution of rWBL is shown in Figure 5. The obligate interfaces have lower average rWBLs than the non-obligate interfaces (also see row 8 of Table 1), although their difference is not very significant, with a p-value of 0.0541, as shown in Table 2. However, the crystal packing interfaces have significantly higher rWBL (p-value: 2.6622 × 10^-5^) than the obligate or non-obligate interfaces, indicating a heavier water exclusion in the formation of biological interfaces.

**Figure 6:**
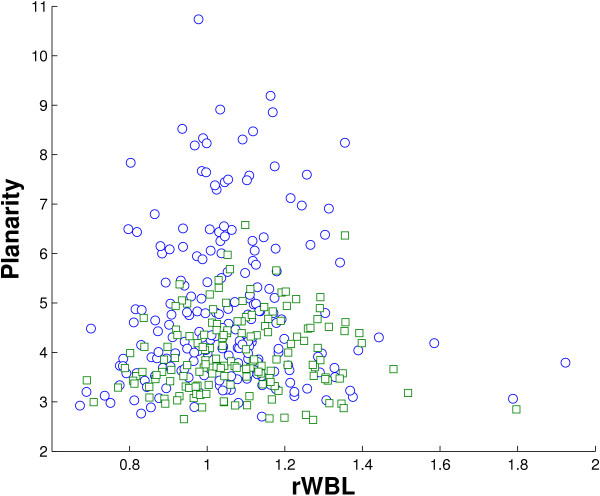
**Relation between rWBL and planarity**. The figure shows a scatter plot of rWBL versus planarity in obligate (blue, circle marker) and non-obligate (green, square marker) interfaces. A few interfaces are observed with very high rWBL and low planarity. These interfaces are extremely dry with very few interfacial water molecules. Their rWBL is not very significant.

One may expect that interfaces with a higher rWBL are more twisted, as twisted interfaces are capable of accommodating more water molecules in their core, with higher wetness and higher rWBL. We investigated the relationship between interface wetness and planarity, but no significant correlation was found. In fact, the correlation coefficients between wetness and planarity are 0.10 and 0.12 for obligate and non-obligate interfaces, respectively. For rWBL, although its correlation coefficient with planarity is even lower than that of wetness, some interesting observation is found. In Figure 6, a scatter plot of rWBL versus planarity in biological interfaces is shown. It can be observed that, when water molecules are strongly excluded (low rWBL, < 0.9), the corresponding interfaces are usually very flat. This suggests that being planar is usually a necessary condition for an interface to exclude its water. However it is not sufficient, as many flat interfaces with a high rWBL were also observed.

Recall that the (negative) correlation between wetness and crystal structure resolution is stronger when the burial level becomes shallower. Thus the wetness of the outer rims of interfaces is more likely to be underestimated than that of the cores. This means that the increase in wetness from core to rim is affirmatively reliable in spite of the different water information quality at different burial levels.

To better understand the influence of water information quality unevenness, we divided the interfaces into three groups according to their level-wise wetness trend: strictly dry-core-wet-rim interfaces, strictly wet-core-dry-rim interfaces, and other interfaces. Strictly dry-core-wet-rim interfaces are referred to as those interfaces whose level-wise wetness increases monotonically from core to rim, while strictly wet-core-dry-rim interfaces are those interfaces whose level-wise wetness decreases monotonically from core to rim. We found, as expected, strictly dry-core-wet-rim interfaces are much more abundant than strictly wet-core-dry-rim interfaces. Over the obligate, non-obligate, and crystal packing interfaces in the data set, there are 87, 83, and 342 strictly dry-core-wet-rim interfaces but only 17, 26, and 124 strictly wet-core-dry-rim interfaces respectively. The strictly wet-core-dry-rim interfaces suffer more from the bad resolution and hence from the bad water information quality. The average resolution for strictly dry-core-wet-rim obligate, non-obligate and crystal packing interfaces are 1.98 Å, 2.18 Å and 2.11 Å, respectively, while the average resolution for strictly wet-core-dry-rim obligate, non-obligate and crystal packing interfaces are 2.35 Å, 2.29 Å and 2.16 Å, respectively (p-values of one-sided difference test: 0.0015, 0.1037 and 0.0403, respectively). This indicates that some water molecules in the rim of the interfaces are not reported and hence the actual wetness of these rims are underestimated, resulting in an overestimate of the number of strictly wet-core-dry-rim interfaces. Nevertheless, there are some high resolution strictly wet-core-dry-rim interfaces. In our data set, there are 4 obligate and 5 non-obligate interfaces that are strictly wet-core-dry-rim interfaces with a resolution better than 2.0 Å. As they are not abundant, we refer them as counterexamples to the dry-core-wet-rim hydration pattern.

**Figure 7:**
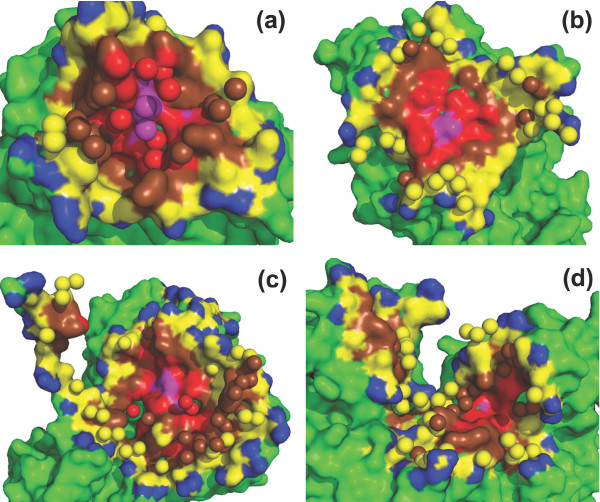
**Examples of interfacial water arrangement**. A counterexample to the dry-core-wet-rim pattern: (a) a yeast triosephosphate isomerase dimer interface ([PDB:1YPI], resolution: 1.90 Å, wetness: 0.044, rWBL: 1.304, level-wise wetnesses from core to rim, similarly hereinafter: 0.077, 0.072, 0.053 and 0.020) and three cases of dry-core-wet-rim water arrangement patterns: (b) a DTDP-glucose 4,6-dehydratase dimer interface ([PDB:1BXK], resolution: 1.90 Å, wetness: 0.059, rWBL: 0.818, level-wise wetnesses: 0.0, 0.0, 0.046, 0.106), (c) an aspartate aminotransferase dimer interface ([PDB:1AJS], resolution: 1.60 Å, wetness: 0.049, rWBL: 1.034, level-wise wetnesses: 0.0, 0.029, 0.058, 0.066) and, (d) an interface between a protein biosynthesis elongation factor eEF1A and its exchange factor eEF1Balpha ([PDB:1 F60], resolution: 1.67 Å, wetness: 0.070, rWBL: 0.935, level-wise wetnesses: 0.0, 0.0, 0.066, 0.112). One side of the interaction partner is shown in surface, with non-interface part colored green and the nested-rings of interface colored according to burial level: *O*_0_, *O*_1_, *O*_2_, *O*_3 _and *O*_4 _are colored magenta, red, brown, yellow and blue, respectively. Interfacial water is shown in spheres.

A counterexample, the yeast triosephosphate isomerase (TIM) dimer interface, is shown in Figure 7(a). In this protein binding interface, the rim is not rich of water molecules, while the core is occupied by a cluster of water molecules. The rWBL of this interface is extremely high (1.304), and the core is the wettest place in this interface. The binding between the two subunits of TIM into a dimer is important as the enzyme is only active in its dimer form [24]. In fact, human TIM deficiency is a rare disease that causes chronic hemolytic anemia and neuromuscular disorders in children [25]. Although it is not a strictly wet-core-dry-rim interface, the human TIM dimer interface is similar to yeast TIM dimer interface, with a very high rWBL (1.282). The most frequent mutation that leads to TIM deficiency, E104D, is in the interface. It is believed that the mutation disrupts the the network formed by interfacial water molecules, then weakens the binding between the two subunits, and thus reduces the activity of the enzyme [26].

Three examples of dry-core-wet-rim interfacial water topological arrangements are presented in Figures 7(b), (c) and 7(d). In the DTDP-glucose 4,6-dehydratase dimer interface shown in Figure 7(b), a large desolvated interface core is observed with rings of gradually increasing water molecules distributed towards to the rim of the interface. In another obligate interface in the aspartate aminotransferase shown in Figure 7(c), more water molecules are observed than in the first example, and several of them penetrate into the core of the interface; yet the amount is not as abundant as that observed in the rim. A twisted non-obligate interface between eEF1A and eEF1Balpha is shown in Figure 7(d). It also shows a dry-core-wet-rim water topology, with a higher wetness than the first two examples. In these three cases, their level-wise wetness goes up progressively from core to rim, being strictly dry-core-wet-rim interfaces.

## Function and interfacial water arrangement

Interfacial water enrichment and organization are different in different functional groups of interfaces. We have manually examined the non-obligate interactions in our data set. Here we describe three types of them, enzyme-inhibitor interactions antibody-antigen interactions, and interactions containing shared hub proteins.

### Enzyme-inhibitor interfaces

There are 42 enzyme-inhibitor interfaces in our data set, accounting for about 25% of the total non-obligate interfaces. All of them are hydrolase-inhibitor interfaces, except one cyclin A-cyclin-dependent kinase 2 interaction [PDB:1JSU] and one Cell division protein kinase 2 [PDB:2CO5]. These enzyme-inhibitor interfaces are of medium wetness (mean: 0.042) and relative low rWBL (mean: 1.042) on average. However, the water topological arrangements within this type of interfaces are extremely heterogeneous. The interfaces between proteases (Enzyme Commission Number: 3.4.-.-) and their inhibitors are significantly drier and with lower rWBL than the other enzyme-inhibitor interfaces; see Table 4. The non-protease-inhibitor interfaces are very wet with the water deeply buried. Their wetness and rWBL are nearly the same as those of crystal packing interfaces.

**Table 4:** Difference between protease-inhibitor and other enzyme-inhibitor interfaces.

Property	No. of interfaces	wetness	rWBL	polarity
Proteases-inhibitor	29	0.036	1.005	0.390
Other enzyme-inhibitor	13	0.054	1.125	0.378
p-value	-	0.002	0.004	0.075

Comparison between protease-inhibitor interfaces and other enzyme-inhibitor interfaces. The statistical significance is tested by one-sided Mann-Whitney U test

Inhibitors usually bind to the active site of an enzyme to block the access to its substrate. Proteases are enzymes that are capable of hydrolyzing peptide bonds. As most inhibitors of proteases are proteins, one mechanism for an inhibitor to avoid being hydrolyzed by the binding protease is to achieve a tight binding between the inhibitor and the enzyme so that water, which is needed in the hydrolysis reaction, is blocked from reaching the active site [27,28]. Thus it is functionally important that the water molecules are excluded from the active site in protease-inhibitor interfaces, resulting in their low wetness. Moreover, the active site is usually located at the center of an interface; thus preventing water from accessing it generally reduces the burial level of water molecules and hence reduces the rWBL, making protease-inhibitor interfaces perfect dry-core-wet-rim interfaces.

**Figure 8:**
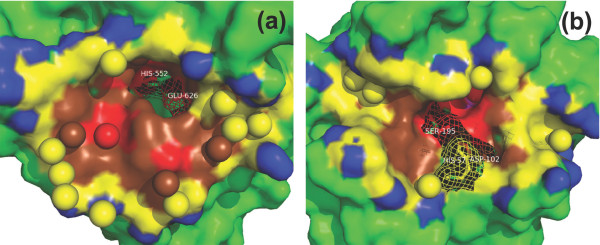
**Two cases of protease-inhibitor interfaces**. (a) The interface between a carboxypeptidase a2 (EC number: 3.4.15.1) and a metallocarboxypeptidase inhibitor ([PDB:1DTD], resolution: 1.65 Å, wetness: 0.055, rWBL: 0.998). (b) The interface between a beta-trypsin (EC number: 3.4.21.4) and its inhibitor ([PDB:2PTC], resolution: 1.9 Å, wetness: 0.029, rWBL: 0.954). The figures only show the enzyme part (in surface) and the interfacial water (in spheres). Non-interface part is colored green. In (a), there are 4 layers of nested-rings in interface: *O*_0 _(red), *O*_1 _(brown), *O*_2 _(yellow) and *O*_3 _(blue). In (b), there are 5 layers of nested-rings: *O*_0 _(magenta), *O*_1 _(red), *O*_2 _(brown), *O*_3 _(yellow) and *O*_4 _(blue). Active site residues [29] are shown in sticks and mesh.

Figures 8(a) and 8(b) show two examples, a wet one and a dry one, of protease-inhibitor interfaces. Both structures have a resolution better than 2.0 Å. It can be noted that, no matter how wet an interface is, water molecules cannot access to its active site residues, which reside at the core of the interface [29]. In both cases, a pocket is observed in the enzyme part, where the inhibitors can anchor deeply into the enzymes to obtain a tight binding. In the wetter interface in Figure 8(a), the pocket is the place where the active site residues are located, thus the pocket is dry with no interfacial water molecules observed inside. In the drier interface in Figure 8(b), the active site residues are not in the pocket; water molecules are observed in the pocket in this case. We should emphasize that, anchoring into this binding pocket shown in Figure 8(b) is very important for the inhibitor to bind tightly with the enzyme (beta-trypsin). The mutation of the anchor residue in the inhibitor (LYS15) into alanine changes the binding affinity dramatically by a *ΔΔG *of about 10 kcal/mol [30], a much bigger *ΔΔG *value than those of hot spot residues without surrounding water molecules. The contrast between the two figures clearly indicates that water molecules may be used to strongly reinforce the binding even in a very important site as long as the function of the binding is preserved.

### Antibody-antigen interfaces

There are 10 antibody-antigen interfaces in the data set. They are very wet with an average wetness 0.047. If only crystal structures of resolution better than 2.0 Å are considered, the average wetness becomes 0.064. Their average rWBL is only 1.037, lower than the average rWBL of all the non-obligate interfaces in the data set. The major difference between antibody-antigen interactions and other non-obligate interactions is that antibody and antigen are poorly related in evolution yet their binding is still of very high affinity and specificity.

This extraordinary requirement for both high binding affinity and specificity has resulted in a specific water distribution topology in antibody-antigen interfaces. Polar and charged residues are often used in antibody-antigen interfaces to enhance the binding specificity. These residues are capable of forming hydrogen bonds and salt bridges; and the electrostatic distribution on antigen and antibody binding sites can selectively determine to which they will bind [31]. This leads to a high hydrophilicity at the interface. In order to achieve high binding affinity at the same time, the hydrogen bonds and salt bridges are usually networked through interfacial water molecules [31,32], which in turn elevates the wetness of the interface.

**Figure 9:**
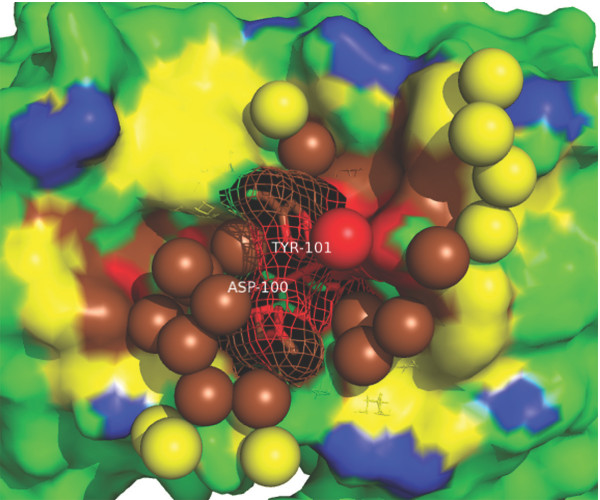
**An example of antibody-antigen interface**. The interface between an anti-hen egg white lysozyme antibody D1.3 and a hen egg white lysozyme ([PDB:1VFB], resolution: 1.8 Å, wetness: 0.083, rWBL: 1.143). Only the antibody part (in surfaces) and interfacial water molecules (in spheres) are shown. *O*_0_, *O*_1_, *O*_2_, *O*_3 _and non-interface are colored red, brown, yellow, blue and green, respectively. Two residues contribute more than 3.0 kcal/mol [33] are highlighted in mesh and sticks.

Figure 9 shows an antibody-antigen interface between an anti-hen egg white lysozyme antibody D1.3 and a hen egg white lysozyme. This interface is the wettest antibody-antigen interface in the data set; yet we still observed a dry-core-wet-rim water distribution topology. There is a tier of water molecules near the edge of the interface and a cluster of water penetrating into a deeper level to shape the binding site by filling a pocket. There are two residues in this interface, TYR101 and ASP100, that contribute significantly more than other residues to the binding free energy [33]. As shown in this figure, water molecules are crowded around these two residues, but these two residues' ability to contact directly with the antigen is not disturbed.

### Interfaces involving hub proteins

Some proteins can interact with many different partners, and maintaining many different functions. These proteins are typically called "hub" proteins. We investigated the water distribution topology of hub proteins by using the "shared proteins" proposed by Keskin and Nussinov [34]. Similar binding sites of these shared proteins are observed to bind with different partners. In protein-protein interaction networks, these proteins are of large connectivity. In terms of structure, these interfaces are of smaller size with larger gap between the two partners, and their shape is flatter.

In our non-obligate interface data set, 10 are also reported in [34] as this kind of interface (Type 3 as in [34]). The average wetness of them is 0.036, insignificantly lower than the overall wetness of non-obligate interfaces, which is, however, unexpected as interfaces containing shared proteins are believed to have more water molecules to bridge inter-protein contacts [34]. Moreover, their rWBL is very low (mean: 0.992), significantly lower than other non-obligate interfaces (p-value: 0.021, one-sided Mann-Whitney U test). It seems that water exclusion is very important for them.

**Figure 10:**
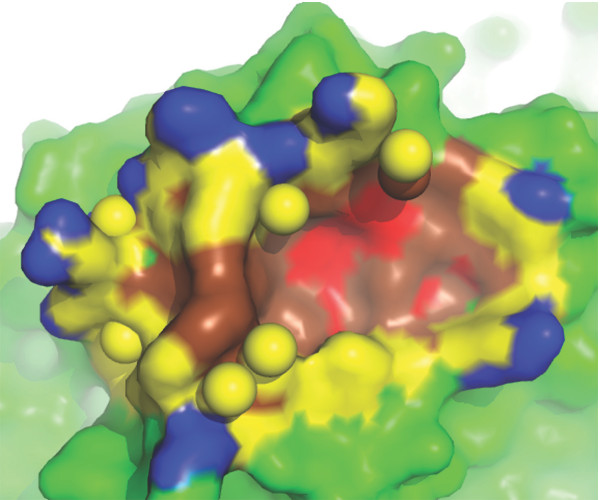
**An example of interface containing a hub protein**. The interface between a transducin with a cGMP phosophodiesterase ([PDB:1FQJ], resolution: 2.02 Å, wetness: 0.032, rWBL: 0.869). Only the transducin (in surfaces) and interfacial water molecules (in spheres) are shown. *O*_0_, *O*_1_, *O*_2_, *O*_3 _and non-interface are colored red, brown, yellow, blue and green, respectively.

Figure 10 shows an example--the binding site of a transducin with cGMP phosophodiesterase (PDE). Transducin is an important G protein in vertebrate phototransduction cascade. The connectivity of this protein is 30 according to the MINT database [34,35]. It is activated by the G-protein-coupled receptor rhodopsin after the the receptor is activated. After that it binds to and activates PDE to enable downstream reactions. There are only 7 water molecules in this interface and the dry-core-wet-rim pattern is again observed. Its rWBL is extremely low (0.87). One possible reason of why the core of this interface is so dry is the transient nature of the binding. The association and disassociation between transducin and PDE are triggered by upstream and downstream signals, and the binding site is veiled when it is not active [36]. The hydrophobic and dry core may reduce the energy barrier of these processes as there is less solvation and desolvation of the binding site. However, detailed and systematic experimental or computational analysis is required to uncover the dynamics of these processes.

## Discussion

It is widely known that exposed protein surfaces directly accessible to bulk solvent are dramatically different from the interiors of protein interfaces [37]. We also find that the interior of protein interface is not the same everywhere in terms of wetness, water-detectability or polarity. Among the reasons for this unevenness, the distance to the bulk solvent--i.e. burial level--is an important one. As discussed earlier, if the interface is organized into rings of residues from its core to the rim, the properties of the rings are different. This reminds us of the famous "O-ring" theory [38,39]. The "O-ring" theory suggests that there is a cluster of residues residing at the core of an interface, contributing most to the binding free energy, while other interfacial residues surround them in a ring-like manner to protect them from the bulk solvent. Our results suggest that there are indeed nested rings of residues in a protein binding interface, progressively growing from the center to the rim of the interface, showing a level-wise pattern. Moreover, the core of an interface is sheltered from water molecules by several rings of atoms, the desolvation power of which increases when one gets deeper into the interface.

**Figure 11:**
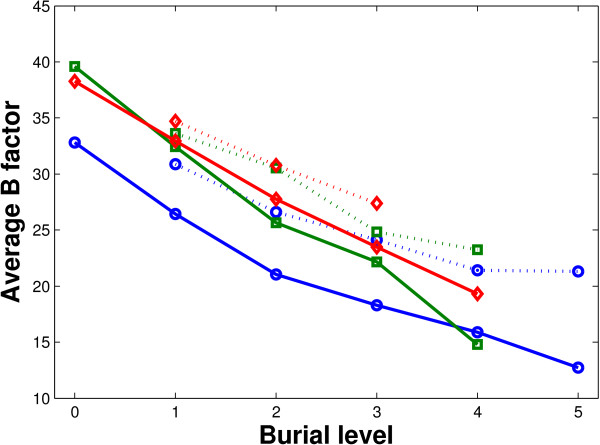
**Relation between burial level and B factor**. Relation between burial level and B factor. The figure shows the average of B factor of water molecules (dotted lines) and non-water interfacial atoms (solid lines) in our data set in obligate (blue, circle marker), non-obligate (green, square marker) and crystal packing (red, diamond marker) protein-water-protein interfaces at different burial levels. The B factors are averaged within the atoms in each interface first and them averaged among the interfaces.

Actually, the nested rings of atoms in protein binding interfaces are also different in their mobility, which can be observed through a level-wise investigation of the B factors. In Figure 11, the average B factors at different burial levels are shown. It can be observed that deeply buried part possesses higher B factors--not only interfacial residues follow this trend, but interfacial water molecules also show such a layered pattern. This indicates that interfacial water molecules in the internal rings are indeed "trapped" by the outer rings of atoms.

The role of water molecules may also be different in different levels of the interface. One of the most important roles of water in protein binding interfaces is bridging the inter-protein contacts by hydrogen bonding with both sides. Specifically, interfacial water molecules prefer to make donor-water-donor or acceptor-water-acceptor hydrogen bond bridges, where the two groups are not complementary to each other originally [40]. We investigated the hydrogen bonds formed by interfacial water molecules at different burial levels (using HBPLUS [41]). The percentage of non-complementary interface hydrogen bond bridges at different burial level is shown in Figure S1 (see Additional file 1). Although fluctuation is observed for transient interfaces, for obligate and crystal packing interfaces, it is observed that deeply buried water molecules are more likely to mediate non-complementary hydrogen bonds.

These observations suggest that protein interfaces do not simply follow a hot spot/O-ring dichotomy. Rather, a protein binding interface is subject to a progressive change in the physicochemical properties from core to rim.

According to the "O-ring" theory, the energy contribution of hot spots in the core is much stronger than the outer ring in the rim. We believe that the energy importance is growing progressively from rim to core, ring by ring. A direct correlation between the energy and burial level can be seen from the Generalized Born model [42] of solvation free energy, in which the atoms are characterized with an effective Born radius. Similar to burial level, the effective Born radius of an atom generally reflects how deep the atom is buried in the solute. However, it is set as a constant in practice. The electrostatic energies also seem to be related to burial level, as the dielectric constant of water is different from that of protein interior. The dielectric constant of water is around 80 [43], while the dielectric constant of protein interior is roughly in the range between 1 and 20 [44]. In energy functions, this difference is considered in a very rough manner, previously. For example, in the FoldX energy function [45], the dielectric constant is linearly scaled from 8 to 80, according to the volumes of the nearby atoms within a distance of 6 Å. There is no further differentiation when atoms are more than 6 Å underneath the surface.

In our previous work [20], we proposed a hot spot prediction model based on the burial level of residues. We found that the average burial level of the atoms in a residue has a positive correlation with the *ΔΔG *caused by alanine mutation with a coefficient of 0.4588. Thus, we believe that incorporating burial level to energy functions explicitly or implicitly will increase the accuracy of binding free energy and hot spot prediction.

We also note that the water distribution topology is different between obligate and non-obligate interfaces, and also between biological and crystal packing interfaces. This encourages us to perform interface classification by taking interfacial water into consideration. For other applications, for example, protein docking, adding water into the model has been already proved to be useful [12]. The general dry-core-wet-rim distribution topology may also be considered in this kind of application to understand a modeled binding interface, or a real binding interface.

## Conclusion

We have studied level-wise water distribution profiles of protein interfaces using a tripartite graph model of protein binding interfaces, i.e., protein-water-protein interfaces. The water arrangement in biological interfaces can be distinguished from that in crystal packing interfaces in different ways such as higher wetness and lower relative water burial level. Differences between obligate and non-obligate interfaces are also observed, yet they are not as significant as those between biological and crystal packing interfaces. Water molecules are generally organized in a dry-core-wet-rim hydration pattern in an interface, suggesting that the core of an interface is protected incrementally by rings of progressively desolvated atoms. We have also conducted an analysis on the water arrangements in different functional groups of protein interfaces. It turns out that the water distributions are subject to the function of the interfaces.

## Methods

### Data set

Our set of obligate and non-obligate interactions are taken from a few previous works. The obligate interactions include those obligate interactions used by Mintseris and Weng [46] and Zhu *et al. *[18], as well as those homodimeric proteins used by Ponstingl *et al. *[15] and Bahadur *et al. *[17]. Our non-obligate interactions include those protein complexes used by Bahadur *et al. *[17], transient interactions used by Mintseris and Weng [46] and non-obligate interactions used by Zhu *et al. *[18]. Crystal packing interactions are collected from the Protein Data Bank (PDB) [47] by taking those interfaces between two chains that are from different biological assemblies according to "REMARK 350". For a protein complex, if another version of the PDB entry with a better resolution (a smaller resolution value) is available, only the better one is used in this work. Redundancy is removed by using a sequence similarity threshold of 30%. That is, if the sequence similarities of any two chains, each from one side of the interaction, with a chain pair from another interaction are both larger than 30%, one of the interfaces is removed. To guarantee the quality of water information, interfaces whose PDB structure contains less than 20 reported water molecules or whose oxygen atoms of water are less than 1% of all the heavy atoms are eliminated. If any chain of an interface requires coordinate transformation, the corresponding interface is removed. Interfaces with less than 100 heavy atoms or have no interfacial water molecules are also eliminated. We removed interfaces with no water--there are only a few such cases--is the reason that it is hard to define the water burial level (WBL, defined later) of such interfaces.

This process results in a total of 206 obligate interactions, 160 non-obligate interactions and 522 crystal packing interactions in our data set. Complete lists of these interfaces are available in Tables S1-S3 (see Additional file 1). It should be noted that the "REMARK 350" in a PDB header is not always correct. However, we believe that such cases are not abundant in this relatively large data set [48,49]. The conclusions we make are hence reliable.

### Construction of atomic contact graphs and protein-water-protein interfaces

We distinguish immobilized water molecules and exposed water molecules in a protein complex by an iterative procedure. First, the solvent accessible surface area (SASA) of the atoms is calculated. Water molecules with SASA larger than 10 Å^2 ^are removed. Then SASAs are calculated again based on the updated structure. This procedure is repeated until there is no water molecule with SASA larger than 10 Å^2 ^in the structure. We refer to the removed water molecules as exposed water molecules and those remaining in the structure as immobilized or buried water molecules.

An atomic contact graph is built based on the structure resulting from the removal of exposed water molecules. The nodes of the graph are atoms and the edges are contacts between atoms. Two atoms are defined to be in contact if (i) they share a Voronoi facet and (ii) their distance is less than their radius plus 2.75 Å, which is the diameter of a water molecule. Two residues are defined to be in contact if there is at least one pair of atoms, one from each residue, that are in contact. The nodes in the atomic contact graph are labeled as "exposed" or "buried" based on their SASA with a threshold of 10 Å^2^. A pseudo node that represents the bulk solvent is added into the graph; this node is directly connected to all the exposed atoms.

The atomic/residue contact graph of a protein complex is denoted by *G *= <*V*, *C *>, where *V *is the set of atoms/residues and *C *⊆ *V *× *V *is the set of contacts. Water molecules in *G *are denoted by the subset *V_W_*. The interfacial water *V_IW _*in the interface between *V_A _*and *V_B _*(*V_A_*, *V_B _*⊆ *V*) is defined as:

(1) $$V_{IW}=\left\{w\in V_{W}|\;\exists v_{a}\in V_{A},v_{b}\in V_{B}:\left(v_{a},w\right),\left(v_{a},w\right)\in C\right\}$$

Interfacial contacts are then defined as:

(2) $$C_{I}=C\cap \left(\left(V_{A}\times V_{B}\right)\cup \left(V_{A}\times V_{IW}\right)\cup \left(V_{B}\times V_{IW}\right)\right)$$

Our tripartite model of protein-water-protein interfaces is defined as the edge-induced subgraph *G_I _*of *G*:

(3) $$G_{I}=G\left[C_{I}\right]$$

We use *V_IA _*and *V_IB _*to denote the interfacial atoms/residues from chain A and B respectively. Our model of protein interfaces can capture those water molecules that immediately bridging the two parts, i.e. water molecules that forming protein-water-protein contacts. That's why we name interfaces under our model protein-water-protein interfaces. We do not consider higher order interfacial water bridges, such as protein-water-water-protein contacts. We believe they are less important and less abundant. More details about the Voronoi facets and the initial idea of the tripartite model of protein binding interfaces can be found in our earlier work [20].

### Calculation of wetness

Suppose *O *is a protein-water-protein interface, we denote its atom-level tripartite graph as

*O *= <*V_IA_*(*O*) ∪ *V_IB_*(*O*) ∪ *V_IW_*(*O*), *C_I_*(*O*) >, where *V_IW _*is the set of oxygen atoms of interfacial water molecules.

The wetness of *O *is defined as:

(4) $$wetness\left(O\right)=|V_{IW}\left(O\right)|/|V_{IA}\left(O\right)\cup V_{IB}\left(O\right)\cup V_{IW}\left(O\right)|$$

where |*X*| is the cardinality of set *X*.

The burial level of an atom *a*, denoted *BL(a)*, in a given protein complex is defined as the length of its shortest path to the nearest exposed atom in the associated atomic contact graph. It is equal to the length of its shortest path to the pseudo node minus one. The average burial level of all water oxygen atoms in *O*, denoted by WBL(*O*), is calculated by:

(5) $$\text{WBL}\left(O\right)=\underset{a\in V_{IW}\left(O\right)}{\sum }\;BL\left(a\right)/|V_{IW}\left(O\right)|$$

The size of an interface *O *is the number of interfacial atoms, including atoms of the amino acids from both sides and the oxygen atoms in the interfacial water molecules, namely |*V_IA_*(*O*) ∪ *V_IB_*(*O*) ∪ *V_IW_*(*O*)|.

The relative water burial level describes in general how deep the water molecules are buried with respect to the average interface burial level. It is defined as:

(6) $$\text{rWBL}\left(O\right)=\frac{\text{WBL}\left(O\right)}{\sum_{a\in O}\;BL\left(a\right)/|V_{IA}\left(O\right)\cup V_{IB}\left(O\right)\cup V_{IW}\left(O\right)|}$$

The level-wise wetness is the proportion of water oxygen atoms over all atoms at a given burial level *i*:

(7) $$wetness^{i}\left(O\right)=|V_{IW}^{i}\left(O\right)|/|V_{IA}^{i}\left(O\right)\cup V_{IB}^{i}\left(O\right)\cup V_{IW}^{i}\left(O\right)|$$

We also define the overall polarity as well as the level-wise polarity of an interface as the proportion of polar atoms, counting O, N and S atoms as polar atoms.

The planarity of an interface is defined as root mean square deviation of non-water interfacial atoms from the least-squares plane of them [6].

### Correlation coefficient

The correlation coefficient between two random variables *X *and *Y *is calculated as the Pearson correlation coefficient:

(8) $$r=\frac{\sum_{i=1}^{n}\;\left(X_{i}-\bar{X}\right)\left(Y_{i}-Ȳ\right)}{\sqrt{\sum_{i=1}^{n}\;{\left(X_{i}-\bar{X}\right)}^{2}}\sqrt{\sum_{i=1}^{n}\;{\left(Y_{i}-Ȳ\right)}^{2}}}$$

Here, $\bar{X}$ is the mean of *X *and *n *is the sample size.

## Competing interests

The authors declare that they have no competing interests.

## Authors' contributions

ZL and JL discussed and designed the experiment. YH and JL supervised the project; YH and LW participated in the analysis; ZL drafted the manuscript; All authors revised and approved the manuscript.

## Supplementary Material

Additional file 1 **One figure and three tables are contained in this file**. The figure is about the hydrogen binding bridges. The three tables are the lists of all interfaces used in this paper, along with their properties. Click here for file
