# Supplementary material for: Progressive dry-core-wet-rim hydration trend in a nested-ring topology of protein binding interfaces
Source: BMC Bioinformatics. 2012 Mar 27;13:51. doi: 10.1186/1471-2105-13-51 (PMC3373366; doi:10.1186/1471-2105-13-51)
Supplement: Additional file 1 — One figure and three tables are contained in this file. The figure is about the hydrogen binding bridges. The three tables are the lists of all interfaces used in this paper, along with their properties. [file 1471-2105-13-51-S1.PDF]

Progressive dry-core-wet-rim hydration trend in a nested-ring topology of protein binding interfaces
Supplementary materials

1 Supplementary Figures

Figure S1 - Proportion of donor-water-donor and acceptor-water-acceptor inter-protein hydrogen bonding bridges at different burial levels.

The percentage of non-complementary donor-water-donor and acceptor-water-acceptor (not donor-water-acceptor) inter-protein hydrogen bonding bridges in obligate (blue, circle marker), non-obligate (green, square marker) and crystal packing (red, diamond marker) protein-water-protein interfaces.

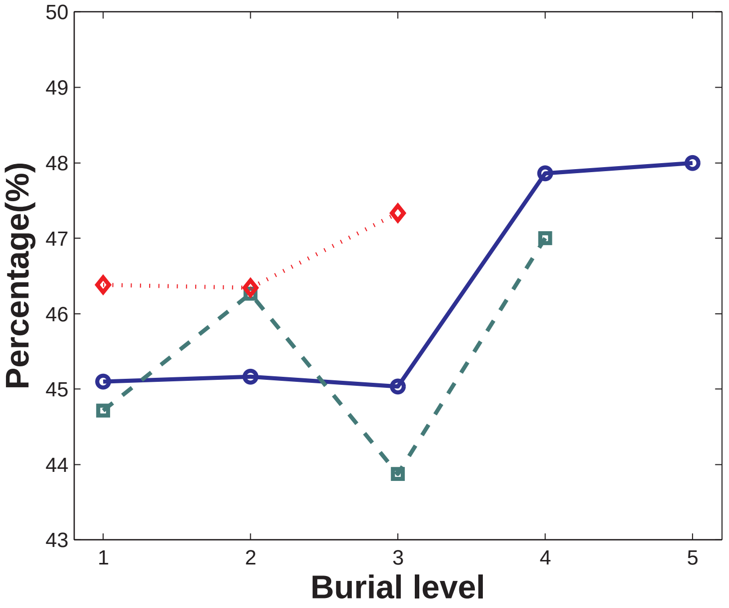

2 Supplementary Tables

Table S1 - Obligate interfaces.

List of all obligate interfaces used.

PDB id	Chains	#atom	#water	wetness	WBL	polarity	rWBL	planarity
12AS	A:B	532	12	0.023	1.167	0.329	0.938	6.134
1A4I	A:B	447	31	0.069	1.968	0.389	1.184	4.074
1AA7	A:B	319	11	0.034	1.455	0.409	1.062	3.521
1AD3	A:B	1168	34	0.029	1.382	0.380	0.999	8.230
1ADE	A:B	896	52	0.058	1.904	0.411	1.134	4.838
1AFW	A:B	812	60	0.074	1.783	0.398	0.932	5.453

1AJ8	A:B	1372	46	0.034	1.696	0.383	1.054	7.496
1AJS	A:B	1085	53	0.049	1.547	0.358	1.034	8.911
1AOR	A:B	391	16	0.041	1.750	0.403	0.966	3.996
1AQ6	A:B	672	20	0.030	1.550	0.363	0.896	3.876
1AT3	A:B	332	1	0.003	1.000	0.366	0.783	3.724
1AUO	A:B	186	1	0.005	1.000	0.335	1.141	2.702
1B34	A:B	227	1	0.004	1.000	0.350	0.775	3.338
1B3A	A:B	249	11	0.044	1.182	0.378	1.078	3.458
1B4U	A:B	622	24	0.039	1.917	0.378	1.178	4.595
1B5E	A:B	744	31	0.042	1.903	0.403	1.091	4.160
1B67	A:B	406	2	0.005	1.000	0.344	0.902	4.559
1B7Y	A:B	1102	11	0.010	1.364	0.364	1.163	9.189
1B8A	A:B	1379	70	0.051	2.114	0.354	1.043	6.550
1B8M	A:B	393	10	0.025	1.500	0.337	1.328	3.687
1B9M	A:B	834	34	0.041	1.206	0.345	0.880	6.150
1BBH	A:B	244	7	0.029	1.000	0.333	0.689	3.202
1BD0	A:B	1183	101	0.085	2.178	0.376	1.037	6.004
1BIS	A:B	496	25	0.050	1.560	0.331	1.029	3.711
1BJN	A:B	688	31	0.045	2.323	0.374	1.286	3.915
1BJW	A:B	876	24	0.027	1.542	0.378	1.045	7.440
1BKP	A:B	708	41	0.058	1.805	0.394	1.105	4.218
1BMD	A:B	521	23	0.044	1.870	0.398	1.184	3.999
1BRM	A:B	885	11	0.012	2.182	0.373	1.355	8.241
1BRW	A:B	293	4	0.014	1.000	0.349	0.736	3.124
1BXG	A:B	291	10	0.034	1.900	0.384	1.307	3.036
1BXK	A:B	454	27	0.059	1.222	0.361	0.818	3.581
1BYF	A:B	256	8	0.031	1.000	0.343	0.751	2.979
1BYK	A:B	390	6	0.015	1.333	0.349	1.040	3.231
1C7N	A:B	670	43	0.064	1.907	0.376	1.054	4.879
1CCW	A:B	386	25	0.065	1.480	0.371	1.084	4.235
1CDC	B:A	934	12	0.013	1.083	0.342	0.938	6.506
1CG2	A:D	423	13	0.031	1.154	0.395	0.992	3.539
1CHM	A:B	1242	85	0.068	2.353	0.372	1.215	7.119
1CLI	A:B	753	25	0.033	1.320	0.368	0.911	4.306
1CMB	A:B	420	5	0.012	1.000	0.313	0.811	4.606
1CNZ	A:B	700	13	0.019	1.231	0.367	0.865	6.794
1COZ	A:B	272	3	0.011	2.333	0.309	1.788	3.062
1CP2	A:B	285	7	0.025	1.857	0.363	1.389	4.042
1CPC	A:B	425	11	0.026	1.636	0.345	1.032	3.430
1CRU	A:B	476	53	0.111	2.170	0.378	1.212	3.641
1CVU	A:B	936	63	0.067	1.635	0.387	1.039	4.497
1D0C	A:B	952	58	0.061	2.052	0.377	1.117	6.257
1DAA	A:B	683	22	0.032	1.727	0.346	1.126	5.774
1DCE	A:B	977	57	0.058	1.316	0.388	1.020	7.380
1DII	A:C	340	15	0.044	1.733	0.357	1.047	3.356
1DJ7	A:B	245	9	0.037	1.222	0.360	1.105	3.985
1DKF	A:B	343	18	0.052	1.278	0.391	0.906	3.716
1DPG	A:B	727	36	0.050	1.417	0.342	0.948	4.079
1DQS	A:B	663	61	0.092	1.393	0.377	0.832	4.857
1DXG	A:B	198	1	0.005	1.000	0.365	0.980	3.186
1DXT	A:B	234	4	0.017	1.250	0.378	0.855	2.888
1E50	A:B	334	10	0.030	1.600	0.395	1.130	3.808
1E9G	A:B	362	22	0.061	1.591	0.338	1.129	3.560
1EEX	A:B	493	42	0.085	1.881	0.388	1.011	4.346
1EEX	A:G	822	40	0.049	1.825	0.363	0.995	4.793
1EFV	A:B	1031	51	0.049	1.804	0.377	1.266	6.175

1EG9	A:B	600	36	0.060	1.889	0.406	1.106	3.870
1EJX	A:C	242	4	0.017	1.250	0.387	1.177	3.258
1EP3	A:B	402	23	0.057	1.609	0.351	1.022	4.328
1ESG	A:B	319	20	0.063	1.350	0.368	0.977	4.031
1EXB	A:E	136	10	0.074	1.200	0.357	1.209	3.740
1EZV	C:F	517	24	0.046	1.750	0.353	1.130	5.318
1EZV	D:H	349	9	0.026	1.222	0.376	0.952	3.493
1F13	A:B	1004	76	0.076	1.605	0.364	1.174	7.762
1F3U	A:B	644	14	0.022	1.000	0.338	0.819	6.436
1F6Y	A:B	395	10	0.025	1.300	0.361	0.876	3.076
1FFU	A:C	382	28	0.073	1.679	0.401	0.886	3.687
1FFV	A:B	772	59	0.076	2.339	0.391	1.000	5.420
1FIP	A:B	376	2	0.005	1.000	0.326	0.816	4.874
1FM0	D:E	303	16	0.053	1.250	0.328	0.942	4.217
1FS0	E:G	670	28	0.042	1.357	0.354	1.123	6.060
1FXW	A:F	331	6	0.018	1.167	0.388	0.988	4.300
1G8K	A:B	602	43	0.071	1.628	0.388	0.959	5.130
1G8T	A:B	319	21	0.066	1.381	0.396	0.941	3.665
1GKA	A:B	225	1	0.004	2.000	0.357	1.923	3.794
1GO3	E:F	575	21	0.037	1.476	0.345	1.171	4.796
1GPE	A:B	524	58	0.111	1.603	0.416	1.056	3.939
1GPU	A:B	1476	76	0.051	2.539	0.386	1.244	6.972
1GPW	A:B	336	14	0.042	1.214	0.410	0.934	3.632
1GUX	A:B	261	5	0.019	1.000	0.313	0.784	3.873
1H18	A:B	694	53	0.076	2.170	0.365	1.179	5.638
1H2R	L:S	1228	88	0.072	2.466	0.371	1.126	4.985
1H2V	C:Z	404	20	0.050	1.350	0.375	0.997	4.047
1H32	A:B	371	23	0.062	1.522	0.356	1.174	5.104
1H8E	A:D	813	69	0.085	1.870	0.390	1.116	4.295
1HCN	A:B	540	10	0.019	1.600	0.381	1.442	4.305
1HFE	L:S	1114	45	0.040	1.467	0.357	0.988	5.885
1HJR	A:C	251	1	0.004	1.000	0.320	0.812	3.030
1HSA	A:B	452	20	0.044	1.150	0.375	0.866	4.645
1HSS	A:B	336	17	0.051	1.706	0.370	1.225	3.202
1HXP	A:B	968	22	0.023	1.318	0.349	0.968	8.186
1ICW	A:B	272	7	0.026	1.000	0.374	0.875	4.007
1IHF	A:B	688	30	0.044	1.500	0.345	1.036	5.504
1IR1	A:S	646	54	0.084	2.093	0.387	1.073	4.463
1ISA	A:B	303	11	0.036	1.636	0.394	1.164	3.346
1IVY	A:B	527	18	0.034	1.389	0.348	0.948	4.810
1JB7	A:B	795	37	0.047	1.595	0.383	1.118	5.852
1JK0	A:B	335	6	0.018	1.667	0.325	1.368	3.227
1JKM	A:B	523	55	0.105	1.818	0.391	1.140	3.522
1JMX	A:G	674	35	0.052	1.800	0.379	1.063	6.477
1JMX	AG:B	661	33	0.050	1.788	0.382	1.030	4.773
1JNR	A:B	1345	72	0.054	2.431	0.383	1.118	8.469
1JUE	A:B	698	38	0.054	1.684	0.330	0.985	5.089
1K28	A:D	254	4	0.016	1.250	0.320	1.142	3.337
1K8K	A:B	204	16	0.078	1.188	0.404	1.106	3.568
1K8K	A:E	296	9	0.030	1.444	0.341	1.082	4.084
1K8K	B:F	300	23	0.077	1.391	0.386	1.001	3.631
1K8K	C:F	433	43	0.099	2.116	0.377	1.053	3.501
1K8K	C:G	232	14	0.060	1.500	0.404	1.081	2.979
1K8K	D:F	645	28	0.043	1.393	0.342	0.954	4.753
1KBA	A:B	147	4	0.027	1.000	0.378	0.967	2.897
1KFU	L:S	766	11	0.014	1.455	0.375	1.177	6.100

1KPE	A:B	548	14	0.026	1.000	0.395	0.701	4.484
1KQF	A:B	868	57	0.066	1.702	0.385	0.974	5.945
1KQF	B:C	600	26	0.043	1.423	0.341	1.053	5.628
1KQP	A:B	819	43	0.053	1.674	0.352	0.903	6.082
1KTD	A:B	906	18	0.020	1.778	0.346	1.342	5.817
1L9B	C:HLM	218	10	0.046	1.800	0.399	1.258	3.267
1LI1	AB:C	1341	60	0.045	1.617	0.368	0.936	8.520
1LTI	AC:DEHFG	632	33	0.052	1.909	0.384	1.146	6.328
1LUC	A:B	665	37	0.056	2.324	0.352	1.132	3.857
1M6P	A:B	358	22	0.061	1.455	0.375	1.192	4.667
1MJG	AB:M	748	38	0.051	1.526	0.365	1.023	4.802
1MKA	A:B	458	12	0.026	1.500	0.341	0.905	4.987
1MSP	A:B	171	2	0.012	1.000	0.361	0.830	2.765
1MXR	A:B	1061	65	0.061	2.215	0.381	1.071	5.018
1NBW	AC:B	326	11	0.034	1.000	0.346	0.908	4.765
1NH2	BC:D	772	21	0.027	1.048	0.346	0.797	6.494
1OH0	A:B	372	16	0.043	1.125	0.410	0.775	3.729
1PGT	A:B	425	26	0.061	1.731	0.386	1.039	4.081
1PNK	A:B	1934	80	0.041	1.688	0.339	1.091	8.304
1POI	A:B	579	21	0.036	1.762	0.380	1.055	4.581
1PP2	L:R	382	16	0.042	1.563	0.355	1.199	4.274
1QDL	A:B	380	6	0.016	1.833	0.382	1.330	3.500
1QFE	A:B	277	5	0.018	1.400	0.316	1.080	3.787
1QFH	A:B	681	27	0.040	1.185	0.382	0.980	4.415
1QHI	A:B	520	26	0.050	2.000	0.332	1.049	3.245
1QIP	A:B	1151	65	0.056	1.508	0.366	0.989	8.332
1QKS	A:B	606	67	0.111	1.970	0.410	1.079	4.095
1QLB	B:C	508	25	0.049	1.600	0.373	0.975	4.830
1QMG	A:B	763	74	0.097	2.189	0.380	1.120	4.973
1QNA	A:B	168	7	0.042	1.429	0.348	1.224	3.119
1QOR	A:B	384	17	0.044	1.294	0.379	0.866	3.525
1QU7	A:B	1002	17	0.017	1.353	0.358	1.108	4.128
1R1T	A:B	501	7	0.014	1.000	0.352	0.893	5.414
1R2F	A:B	543	25	0.046	1.440	0.365	0.965	3.827
1R31	A:B	1373	47	0.034	1.596	0.363	0.884	6.001
1REG	X:Y	172	5	0.029	1.200	0.407	1.214	3.390
1REQ	A:B	1686	90	0.053	1.744	0.356	0.978	10.736
1RFB	A:B	672	9	0.013	1.222	0.297	1.304	6.380
1SBY	A:B	828	54	0.065	1.593	0.331	1.007	6.487
1SES	A:B	580	9	0.016	2.222	0.340	1.290	3.597
1SG0	A:B	655	36	0.055	2.194	0.321	1.110	5.166
1SOX	A:B	515	39	0.076	2.359	0.389	1.295	3.981
1SPP	A:B	238	8	0.034	1.125	0.404	0.845	3.299
1SPU	A:B	2302	142	0.062	2.113	0.387	1.169	8.856
1TBG	A:E	572	3	0.005	1.333	0.359	1.115	4.209
1TC1	A:B	477	25	0.052	1.320	0.363	0.918	3.982
1TCO	A:B	538	19	0.035	1.316	0.301	0.830	4.152
1TJP	A:B	469	27	0.058	1.667	0.364	1.009	4.256
1U2G	A:B	530	26	0.049	1.692	0.393	1.098	5.604
1U5B	A:B	566	26	0.046	1.500	0.378	1.046	6.348
1VCB	A:B	283	3	0.011	1.667	0.332	1.375	3.099
1VFR	A:B	949	34	0.036	1.676	0.366	1.103	7.480
1WTL	A:B	190	2	0.011	1.500	0.266	1.173	3.614
1XG0	A:C	702	52	0.074	1.904	0.378	1.160	5.284
1XSO	A:B	219	5	0.023	1.000	0.369	0.839	3.428
1Y6V	A:B	1270	73	0.057	2.247	0.410	1.105	4.769

1YPH	E:C	826	43	0.052	2.302	0.381	1.313	6.910
1YPI	A:B	503	22	0.044	2.409	0.385	1.304	4.795
2AAI	A:B	486	14	0.029	1.143	0.362	0.941	5.350
2AE2	A:B	428	20	0.047	1.300	0.353	0.871	3.879
2AL1	A:B	565	21	0.037	1.381	0.404	0.895	3.275
2ARC	A:B	258	9	0.035	1.333	0.357	1.103	3.470
2BB9	A:B	457	9	0.020	1.333	0.357	1.110	5.089
2CCY	A:B	232	6	0.026	1.000	0.372	0.792	3.578
2GH5	A:B	1061	69	0.065	2.174	0.353	1.137	4.832
2H6F	A:B	1234	107	0.087	1.991	0.387	0.997	7.641
2HDH	A:B	468	25	0.053	1.640	0.321	1.122	3.759
2HHM	A:B	533	28	0.053	1.714	0.345	1.089	4.206
2I5N	C:HLM	1402	77	0.055	1.935	0.362	1.031	6.433
2JHF	A:B	559	31	0.055	1.548	0.366	1.034	6.252
2LIG	A:B	494	16	0.032	1.375	0.368	1.090	4.209
2LYN	A:B	295	14	0.047	1.500	0.310	1.103	4.176
2MIN	A:B	1443	79	0.055	2.709	0.365	1.257	7.596
2MTA	H:L	467	15	0.032	2.000	0.345	1.184	4.066
2NAC	A:B	1192	62	0.052	1.484	0.376	0.803	7.836
2RSP	A:B	378	6	0.016	1.000	0.417	0.873	4.429
2SCU	A:B	557	24	0.043	1.375	0.386	1.009	6.060
2SPC	A:B	689	11	0.016	1.182	0.348	1.041	4.116
2SQC	A:B	295	21	0.071	1.571	0.383	1.032	3.331
2UTG	A:B	373	8	0.021	2.125	0.312	1.585	4.186
2WE5	A:B	631	41	0.065	1.878	0.407	1.001	3.981
2ZPB	A:B	1198	61	0.051	1.754	0.346	1.108	7.576
3DAP	A:B	742	18	0.024	1.667	0.354	0.986	7.668
3DJQ	A:B	538	18	0.033	1.056	0.373	0.857	3.901
3PCE	A:M	1100	60	0.055	1.917	0.357	1.024	7.292
3SDH	A:B	328	26	0.079	1.731	0.387	1.061	3.241
3TMK	A:B	273	12	0.044	1.000	0.360	0.672	2.926
5RUB	A:B	879	32	0.036	1.969	0.392	1.114	4.672

Table S2 - Non-obligate interfaces.

List of all non-obligate interfaces used. †: PI indicates protease-inhibitor interaction; EI indicates enzyme-inhibitor interface but the enzyme is not a protease; ABAG indicates antobody-antigen interface and; HUB indicates one of the interaction partner is a hub protein.

PDB id	Chains	#atom	#water	wetness	WBL	polarity	rWBL	planarity	remarks†
1A2K	A:D	261	13	0.050	1.462	0.355	1.042	4.350	
1A4Y	A:B	400	15	0.037	1.533	0.421	1.205	4.944	EI
1ACB	E:I	233	6	0.026	1.500	0.366	0.996	3.759	PI
1AK4	A:D	149	2	0.013	1.000	0.361	0.892	2.966	
1AKJ	AB:DE	314	9	0.029	1.000	0.439	1.030	5.460	
1AVA	A:C	476	35	0.074	1.714	0.358	1.190	4.639	EI
1AVG	HL:I	190	2	0.011	1.000	0.388	0.905	3.417	PI
1AVW	A:B	295	8	0.027	1.250	0.401	0.802	3.988	PI,HUB
1AWC	A:B	248	8	0.032	1.375	0.350	1.076	3.689	
1AY7	A:B	193	10	0.052	1.300	0.388	1.004	3.346	EI
1AZZ	A:CD	448	10	0.022	1.300	0.386	0.929	5.375	PI,HUB
1BDJ	A:B	104	1	0.010	1.000	0.437	1.072	2.929	
1BJ1	HL:VW	273	6	0.022	1.167	0.337	0.813	3.369	ABAG
1BRS	A:D	303	18	0.059	1.500	0.368	1.052	4.446	EI
1BUH	A:B	172	2	0.012	1.000	0.312	0.851	3.089	

1BVN	P:T	363	14	0.039	2.286	0.387	1.290	4.896	EI
1C1Y	A:B	217	11	0.051	1.091	0.408	0.970	3.307	
1C4Z	A:D	245	9	0.037	1.444	0.343	1.035	3.688	
1CHO	FG:I	245	9	0.037	1.556	0.386	1.024	3.715	
1CLV	A:I	380	27	0.071	1.963	0.371	1.115	4.758	EI
1CMX	A:B	351	9	0.026	2.222	0.371	1.354	4.432	
1CS4	AB:C	250	3	0.012	1.667	0.389	1.398	4.188	
1CSE	I:E	285	21	0.074	1.429	0.394	0.923	4.351	PI
1CXZ	A:B	252	7	0.028	1.429	0.429	1.304	3.462	
1D09	A:B	214	3	0.014	1.333	0.427	1.102	3.514	
1D2Z	A:B	257	9	0.035	1.333	0.379	1.071	3.722	
1D4X	A:G	371	14	0.038	1.071	0.359	0.810	3.428	
1D5M	A:C	236	7	0.030	1.714	0.397	1.272	3.751	
1DHK	A:B	515	33	0.064	1.879	0.411	1.053	5.973	EI
1DOA	A:B	423	8	0.019	1.125	0.386	0.919	4.939	
1DOW	A:B	364	16	0.044	1.313	0.353	1.062	3.818	HUB
1DPJ	A:B	493	34	0.069	2.118	0.359	1.284	4.673	PI
1DTD	A:B	257	14	0.054	1.429	0.362	0.998	3.161	PI
1DU3	A:DEF	375	3	0.008	1.333	0.430	1.250	4.235	
1DVF	AB:CD	275	17	0.062	1.588	0.395	1.060	3.349	
1DX5	AM:I	255	13	0.051	1.308	0.388	1.101	3.668	
1E6E	A:B	343	11	0.032	1.273	0.413	1.091	5.040	
1E96	A:B	173	4	0.023	1.500	0.373	1.311	3.432	
1EAI	A:C	273	7	0.026	1.571	0.429	1.179	3.859	PI
1EER	A:B	327	16	0.049	1.625	0.354	1.121	3.858	
1EFN	A:B	168	2	0.012	2.000	0.355	1.797	2.849	
1EFU	A:B	533	18	0.034	1.278	0.384	1.100	4.062	
1EFX	ABC:D	208	5	0.024	1.800	0.409	1.352	2.872	
1EMV	A:B	220	6	0.027	1.500	0.379	1.209	3.996	
1ES7	AC:B	333	9	0.027	1.222	0.349	0.917	3.888	
1EUV	A:B	416	23	0.055	1.217	0.420	0.898	4.114	
1EZX	AB:C	110	1	0.009	1.000	0.459	0.940	2.651	PI
1F34	A:B	440	10	0.023	1.500	0.388	1.078	4.628	PI
1F3V	A:B	236	8	0.034	1.375	0.417	1.163	3.403	
1F60	A:B	590	41	0.069	1.366	0.397	0.935	5.053	
1F80	A:E	176	4	0.023	1.250	0.436	1.043	3.009	
1F93	AB:EF	242	1	0.004	1.000	0.394	0.708	2.994	
1FAK	HL:T	591	37	0.063	1.378	0.399	1.111	5.283	
1FFG	A:B	179	7	0.039	1.429	0.349	1.291	3.845	
1FLE	E:I	252	8	0.032	1.250	0.398	0.908	3.645	PI
1FLT	VW:X	303	21	0.069	1.667	0.344	1.305	3.495	
1FNS	A:HL	213	9	0.042	1.111	0.382	0.833	3.608	HUB
1FQJ	A:C	216	7	0.032	1.143	0.340	0.869	3.544	HUB
1G4Y	B:R	312	4	0.013	1.250	0.360	1.266	4.496	HUB
1GAQ	A:B	185	1	0.005	1.000	0.435	1.039	4.295	
1GC1	C:G	337	14	0.042	1.571	0.424	1.024	4.448	
1GCQ	B:C	196	5	0.026	1.200	0.361	0.980	3.611	HUB
1GHQ	A:B	169	10	0.059	1.000	0.396	0.867	3.463	
1GL1	A:I	255	7	0.027	1.857	0.387	1.259	4.484	PI
1GL4	A:B	330	18	0.055	1.500	0.385	0.948	3.743	HUB
1GO4	A:G	391	10	0.026	1.100	0.381	0.996	3.840	
1GOT	A:B	415	18	0.043	1.611	0.390	1.203	5.231	
1GVN	AC:B	510	37	0.073	1.514	0.414	1.025	4.573	
1GZS	A:B	442	19	0.043	1.526	0.371	1.044	3.519	
1H2K	A:S	430	16	0.037	1.313	0.384	0.988	4.111	
1H59	A:B	156	5	0.032	1.000	0.384	1.013	3.573	

1HE1	A:C	392	27	0.069	1.481	0.381	1.015	4.872	
1HEZ	AB:E	157	2	0.013	1.500	0.381	1.273	2.637	ABAG
1HIA	AB:I	283	12	0.042	1.417	0.369	0.889	3.856	PI,HUB
1HWG	A:BC	617	15	0.024	1.400	0.402	1.059	5.682	
1HX1	A:B	293	14	0.048	1.143	0.427	1.043	2.995	
1I2M	A:B	499	28	0.056	1.857	0.439	1.089	4.629	
1I4D	AB:D	252	8	0.032	1.500	0.393	1.174	3.317	
1I7W	A:B	658	24	0.036	1.208	0.371	1.049	5.003	
1IB1	AB:E	375	5	0.013	1.200	0.405	1.030	5.306	
1IBR	A:B	558	26	0.047	1.346	0.387	1.098	6.575	
1ICF	AB:I	309	10	0.032	1.400	0.408	1.023	3.873	PI
1IJK	A:BC	222	4	0.018	1.000	0.385	0.982	3.145	
1IM3	AB:D	231	16	0.069	1.250	0.377	1.132	3.610	
1IOD	AB:G	243	23	0.095	1.304	0.359	1.157	4.409	PI
1IQD	AB:C	306	16	0.052	1.938	0.386	1.341	3.471	ABAG,HUB
1IS8	ABEJCIDH GF:KLOMN	466	10	0.021	1.000	0.336	1.118	3.821	
1JCH	A:B	564	5	0.009	1.600	0.356	1.355	6.365	
1JIW	I:P	344	22	0.064	1.500	0.413	0.992	4.359	PI
1JMA	A:B	199	2	0.010	1.500	0.386	1.315	2.972	
1JSU	AB:C	762	12	0.016	1.167	0.343	1.007	5.173	EI
1JTD	A:B	377	27	0.072	1.778	0.354	1.208	3.748	EI
1JTG	A:B	486	30	0.062	1.800	0.406	1.051	4.392	EI
1JW9	B:D	375	20	0.053	1.450	0.366	1.013	4.603	
1K3Z	AB:D	587	5	0.009	1.200	0.368	1.229	5.076	
1K5D	A:B	655	3	0.005	1.333	0.373	1.313	4.518	
1K5D	A:C	419	21	0.050	1.619	0.402	1.164	3.630	
1KAC	A:B	222	8	0.036	1.125	0.360	0.987	2.889	
1KCG	AB:C	299	9	0.030	1.556	0.362	1.285	4.094	
1KI1	A:B	400	16	0.040	1.438	0.365	1.014	3.691	
1KKL	ABC:H	228	2	0.009	1.000	0.403	0.838	4.116	
1KXP	A:D	572	39	0.068	1.821	0.347	1.178	5.657	
1KXQ	H:A	390	26	0.067	1.500	0.390	1.147	4.915	
1KZY	A:C	191	8	0.042	1.125	0.404	0.972	3.159	
1L0O	AB:C	275	3	0.011	1.333	0.386	1.149	4.529	
1L6X	A:B	224	13	0.058	1.000	0.370	0.818	3.522	
1LFD	A:B	195	12	0.062	1.833	0.399	1.184	3.224	
1LK3	A:HL	280	15	0.054	1.600	0.366	0.991	3.694	ABAG
1LPB	A:B	202	5	0.025	1.600	0.386	1.517	3.181	
1M1E	A:B	486	18	0.037	1.111	0.425	0.994	4.053	
1M2O	AC:B	447	16	0.036	1.438	0.392	1.100	4.713	
1M4U	A:L	112	4	0.036	1.250	0.315	1.346	2.965	
1MBX	A:C	242	10	0.041	1.500	0.371	1.349	3.577	
1NBF	A:D	516	17	0.033	1.824	0.389	1.169	4.831	
1NF5	A:B	215	12	0.056	1.583	0.350	1.044	3.731	
1NMB	N:LH	204	5	0.025	1.400	0.387	1.103	2.934	ABAG
1O6S	A:B	567	50	0.088	1.420	0.398	0.948	4.989	
1O94	AB:CD	207	18	0.087	1.278	0.392	1.140	3.730	
1OSP	LH:O	266	17	0.064	1.235	0.406	0.975	3.331	ABAG
1PDK	A:B	415	4	0.010	1.500	0.355	1.157	4.681	
1PPF	E:I	236	8	0.034	1.000	0.373	0.690	3.436	PI
1QAV	A:B	234	7	0.030	1.429	0.352	1.161	2.665	
1QKZ	A:HL	216	12	0.056	1.167	0.382	0.923	3.032	ABAG
1QO0	A:DE	355	25	0.070	1.720	0.382	1.139	3.574	
1SBB	A:B	156	1	0.006	1.000	0.361	0.929	3.113	
1SMF	E:I	164	4	0.024	1.750	0.400	1.167	3.050	PI

1STF	E:I	301	12	0.040	1.000	0.367	0.788	3.683	PI
1TAB	I:E	226	5	0.022	1.400	0.407	0.939	3.521	PI
1TBR	H:R	479	9	0.019	1.667	0.409	1.192	5.211	PI
1TGS	I:Z	270	8	0.030	1.375	0.378	0.954	4.330	PI
1TMQ	A:B	399	23	0.058	1.913	0.383	1.089	4.392	EI
1TX4	A:B	421	39	0.093	1.615	0.390	1.041	4.319	
1UEA	A:B	363	5	0.014	1.000	0.349	0.836	4.697	PI
1UGH	E:I	335	17	0.051	1.941	0.362	1.232	4.320	EI
1VFB	AB:C	265	22	0.083	1.682	0.399	1.143	3.355	ABAG
1WEJ	F:HL	238	15	0.063	1.000	0.395	0.770	3.288	ABAG
1WWW	VW:X	386	25	0.065	1.720	0.371	1.292	5.116	
1X9M	A:B	296	4	0.014	1.750	0.332	1.480	3.662	
1XDT	R:T	272	7	0.026	1.714	0.374	1.392	4.390	
1YCS	A:B	238	4	0.017	1.000	0.423	0.975	4.187	
1ZBD	A:B	345	3	0.009	1.333	0.354	1.278	3.703	
2AFH	AB:EF	549	42	0.077	1.452	0.398	1.112	4.322	
2BTC	E:I	298	14	0.047	1.571	0.366	1.007	4.088	PI
2C5O	A:B	508	25	0.049	1.600	0.364	1.127	4.348	EI
2GC4	L:K	138	5	0.036	1.400	0.414	1.255	2.739	
2HVK	AB:C	247	10	0.040	1.200	0.384	0.890	3.063	
2JEL	HL:P	253	10	0.040	1.500	0.399	1.040	4.133	ABAG
2MTA	A:HL	208	7	0.034	1.429	0.323	1.109	3.115	
2PCC	A:B	215	18	0.084	1.333	0.421	1.111	3.695	
2PRG	B:C	199	1	0.005	1.000	0.369	0.884	3.608	
2PTC	E:I	243	7	0.029	1.429	0.381	0.954	3.284	PI
2SIC	E:I	290	14	0.048	1.357	0.402	0.920	4.462	PI
2TRC	B:P	647	20	0.031	2.000	0.392	1.356	4.609	
3CX5	E:JK	215	8	0.037	1.500	0.411	1.199	2.680	
3CX5	O:W	163	12	0.074	1.417	0.424	1.160	3.483	
3HB3	AB:CD	264	20	0.076	1.400	0.402	0.965	3.080	
3SGB	E:I	211	5	0.024	1.200	0.408	0.945	3.311	PI
3YGS	C:P	177	6	0.034	1.000	0.409	0.851	3.305	
4HTC	H:I	511	25	0.049	1.720	0.379	1.216	4.174	PI
4SGB	E:I	213	9	0.042	1.556	0.422	1.274	3.528	PI

Table S3 - Crystal packing interfaces.

List of all crystal packing interfaces used.

PDB id	Chains	#atom	#water	wetness	WBL	polarity	rWBL	planarity
1ADJ	A:D	175	1	0.006	1.000	0.431	1.577	3.187
1AFR	E:F	225	12	0.053	1.167	0.408	1.172	4.453
1AIH	B:C	127	3	0.024	1.000	0.492	1.085	2.886
1B6S	A:C	275	7	0.025	1.143	0.399	1.173	4.001
1CA9	A:E	172	10	0.058	1.000	0.401	1.229	5.017
1CBW	D:I	107	6	0.056	1.000	0.356	0.856	3.034
1CNO	C:H	133	6	0.045	1.333	0.370	1.174	3.140
1DD3	B:D	237	5	0.021	1.800	0.306	1.385	4.439
1DJ8	C:E	113	3	0.027	1.000	0.364	1.361	3.232
1DXP	A:B	130	6	0.046	1.333	0.411	1.140	3.041
1E3U	A:D	205	14	0.068	1.357	0.398	1.184	4.125
1E50	A:C	176	3	0.017	1.000	0.393	0.842	3.525
1E5Q	F:G	122	7	0.057	1.143	0.417	1.056	3.067
1EEF	G:M	101	8	0.079	1.375	0.430	1.208	3.049
1EJH	A:C	118	1	0.008	2.000	0.393	2.052	2.599

1ELW	A:B	125	16	0.128	1.375	0.404	1.074	3.440
1F3U	E:G	112	8	0.071	1.000	0.394	1.067	2.538
1F5Q	B:C	122	3	0.025	1.333	0.387	1.261	3.353
1F8S	D:F	225	10	0.044	1.000	0.358	1.042	4.348
1FD3	C:D	112	3	0.027	1.333	0.294	1.224	2.757
1FQJ	B:E	313	11	0.035	1.545	0.391	1.363	4.786
1FSE	A:C	146	7	0.048	1.143	0.410	1.017	2.739
1FX7	B:C	151	9	0.060	1.444	0.401	1.338	3.111
1FXO	B:E	242	19	0.079	1.053	0.444	0.791	3.829
1FYH	A:D	158	11	0.070	1.000	0.395	0.952	3.089
1FYH	B:D	125	7	0.056	1.286	0.364	1.236	2.958
1G5C	A:C	192	4	0.021	1.250	0.324	0.968	2.860
1G5I	A:C	174	9	0.052	1.222	0.473	0.962	3.184
1G8K	E:G	936	91	0.097	1.560	0.420	1.052	5.480
1GEF	A:B	286	21	0.073	1.333	0.392	0.980	4.314
1GJY	B:D	157	2	0.013	1.500	0.361	1.246	2.561
1GK4	A:F	253	25	0.099	1.360	0.408	1.027	2.841
1GQG	B:C	252	19	0.075	1.316	0.386	1.143	3.963
1H21	B:C	101	1	0.010	1.000	0.390	1.098	3.357
1H48	C:D	125	4	0.032	1.250	0.314	1.093	2.667
1H5B	A:D	103	8	0.078	1.000	0.421	1.431	3.729
1HC7	B:D	258	3	0.012	1.000	0.463	1.380	3.601
1HF2	B:C	334	20	0.060	1.300	0.408	1.080	4.312
1HFB	B:F	137	9	0.066	1.444	0.344	1.571	3.489
1HIA	B:Y	153	12	0.078	1.750	0.426	1.319	3.190
1HIW	A:R	191	3	0.016	1.333	0.404	1.472	3.100
1IGQ	C:D	148	14	0.095	1.714	0.366	1.226	3.171
1IV1	C:B	144	11	0.076	1.364	0.346	1.097	2.858
1IZN	B:D	322	32	0.099	1.625	0.407	1.217	4.178
1JC5	C:F	152	1	0.007	1.000	0.404	1.126	2.687
1JOP	A:B	101	2	0.020	1.000	0.434	1.098	2.563
1JRI	B:I	146	2	0.014	1.500	0.438	1.422	3.263
1K8C	A:D	119	5	0.042	1.000	0.377	1.227	3.373
1KPS	B:D	217	15	0.069	1.667	0.421	1.106	3.279
1KQ1	I:R	135	18	0.133	1.556	0.376	1.160	3.484
1KVE	A:C	159	13	0.082	1.308	0.445	1.155	3.342
1KXG	A:F	165	12	0.073	2.083	0.386	1.215	3.258
1KXQ	C:D	291	24	0.082	1.208	0.408	1.116	3.271
1L6L	E:N	430	12	0.028	1.167	0.301	1.088	3.324
1L7D	B:C	180	8	0.044	1.125	0.413	1.089	3.489
1M0D	A:D	114	7	0.061	1.000	0.383	0.966	3.063
1M56	B:I	123	5	0.041	1.400	0.373	1.400	3.113
1M7E	B:C	137	2	0.015	1.000	0.437	1.489	3.357
1MKX	H:K	205	2	0.010	1.000	0.350	1.297	3.543
1MV8	A:C	131	3	0.023	1.000	0.422	1.297	3.166
1MVF	D:E	368	2	0.005	1.000	0.320	0.870	4.788
1MZ8	A:C	132	11	0.083	1.909	0.388	1.120	3.138
1N0T	A:B	167	11	0.066	1.273	0.353	1.174	3.254
1N4M	A:B	369	13	0.035	1.000	0.404	0.927	3.306
1N4P	B:J	171	8	0.047	1.375	0.429	1.125	3.416
1N4P	C:I	158	4	0.025	1.250	0.351	1.091	3.575
1N71	A:D	144	4	0.028	1.000	0.407	1.286	2.735
1N8B	A:D	114	1	0.009	1.000	0.354	1.295	4.893
1NKH	B:D	103	2	0.019	1.000	0.287	0.837	2.453
1NMM	A:C	134	15	0.112	1.200	0.378	1.079	3.043
1NPP	A:C	229	13	0.057	1.000	0.431	1.101	3.227

1NVJ	B:C	169	9	0.053	1.556	0.388	1.179	4.043
1NVM	C:F	185	19	0.103	1.421	0.392	1.027	3.483
1O7A	A:C	273	16	0.059	1.188	0.447	1.175	3.490
1O9K	A:E	291	2	0.007	1.000	0.422	1.054	3.096
1ODB	C:E	136	4	0.029	2.000	0.394	1.177	2.752
1OE0	B:C	159	5	0.031	1.000	0.442	1.144	2.961
1OJ7	A:B	325	29	0.089	1.172	0.395	1.035	3.431
1OK6	E:J	138	8	0.058	1.000	0.385	0.920	3.445
1ORJ	B:D	191	9	0.047	1.222	0.379	1.122	3.157
1OTJ	C:D	315	24	0.076	1.667	0.378	1.082	3.779
1OXK	C:F	158	1	0.006	1.000	0.357	0.859	2.759
1OXK	E:I	142	3	0.021	1.667	0.460	1.337	2.776
1P4A	B:C	151	9	0.060	1.111	0.415	1.173	3.641
1Q23	D:J	304	10	0.033	1.200	0.425	1.271	3.415
1Q52	F:H	190	18	0.095	1.167	0.419	0.920	3.365
1Q8M	A:C	145	4	0.028	1.500	0.383	1.377	2.874
1R0K	B:C	110	7	0.064	1.143	0.447	1.310	3.018
1R0V	A:D	109	5	0.046	1.200	0.365	1.108	3.077
1R17	A:B	277	16	0.058	1.313	0.429	1.112	3.546
1R1U	A:D	158	18	0.114	1.611	0.457	1.102	3.026
1R4C	A:C	162	3	0.019	1.667	0.465	1.216	2.793
1R4W	A:D	123	5	0.041	1.000	0.381	0.842	2.987
1RVG	B:D	157	4	0.025	1.500	0.418	1.463	3.061
1RXC	F:I	170	11	0.065	1.545	0.434	1.307	3.027
1RY9	A:D	209	13	0.062	1.154	0.434	1.031	3.769
1S0Y	C:K	101	8	0.079	1.125	0.419	0.854	2.770
1S4D	E:H	171	7	0.041	1.143	0.451	1.110	3.208
1S7N	A:C	126	11	0.087	1.182	0.417	1.064	3.708
1SYX	B:D	105	3	0.029	1.000	0.353	1.071	2.582
1T5O	A:D	206	20	0.097	1.300	0.371	1.149	3.318
1T70	C:F	222	8	0.036	1.375	0.350	1.110	3.604
1TED	A:C	259	9	0.035	1.111	0.368	1.209	3.603
1TEX	B:C	175	11	0.063	1.182	0.390	1.083	3.458
1TJL	B:J	306	14	0.046	1.000	0.411	0.953	3.926
1TLB	S:U	252	8	0.032	1.125	0.430	1.023	3.035
1U5W	E:G	303	10	0.033	1.200	0.375	0.928	3.409
1U60	A:D	108	7	0.065	1.286	0.416	1.093	2.847
1U9L	A:B	101	7	0.069	1.286	0.415	1.073	3.282
1UAD	B:C	107	7	0.065	1.857	0.480	1.603	2.762
1UFB	A:C	154	10	0.065	1.300	0.396	1.106	3.515
1UTC	A:B	252	8	0.032	1.125	0.459	1.129	4.959
1UUP	A:B	190	3	0.016	1.000	0.358	1.005	3.313
1UVZ	B:C	142	8	0.056	1.125	0.343	1.018	3.296
1UW4	B:D	209	2	0.010	1.000	0.324	1.015	3.405
1UX2	C:F	227	15	0.066	1.200	0.410	1.036	4.469
1V0J	A:C	393	26	0.066	1.731	0.379	1.169	3.396
1V1H	C:F	107	6	0.056	1.333	0.386	1.372	2.684
1V4P	B:C	210	16	0.076	1.313	0.361	1.125	3.800
1V7C	A:D	134	12	0.090	1.250	0.377	1.102	3.489
1VAY	B:G	226	13	0.058	1.615	0.465	1.263	3.859
1VCB	C:K	125	1	0.008	2.000	0.355	1.908	2.799
1VCH	A:C	128	8	0.063	1.500	0.417	1.110	3.398
1VGW	D:F	166	2	0.012	1.000	0.427	0.927	3.530
1VI6	A:D	222	19	0.086	1.263	0.399	0.957	3.242
1VYJ	E:I	198	7	0.035	1.714	0.414	1.342	4.144
1VYT	A:B	166	2	0.012	1.500	0.378	1.682	3.268

1VZ0	F:G	123	8	0.065	1.125	0.426	1.107	3.232
1W9Q	A:B	350	21	0.060	1.429	0.395	1.136	3.285
1WBI	A:G	152	10	0.066	1.400	0.359	1.059	3.416
1WM6	A:F	197	11	0.056	1.545	0.366	1.238	4.020
1WMW	A:D	221	23	0.104	1.957	0.414	1.239	3.154
1WMZ	B:D	235	26	0.111	1.692	0.459	0.977	3.604
1WW7	C:D	116	4	0.034	1.000	0.420	0.906	2.659
1WX5	A:C	206	12	0.058	1.250	0.459	1.030	3.338
1X8D	A:C	118	12	0.102	1.250	0.368	1.031	3.026
1XCC	A:B	159	7	0.044	1.000	0.408	1.013	2.930
1XD5	B:C	168	6	0.036	1.500	0.438	1.377	3.203
1XED	E:F	262	17	0.065	1.471	0.359	1.047	3.143
1XIW	C:F	175	12	0.069	1.167	0.374	0.941	3.125
1XMM	B:D	225	11	0.049	1.364	0.383	0.990	3.371
1XXU	C:D	177	5	0.028	1.600	0.285	1.248	2.442
1Y0G	A:C	109	6	0.055	1.000	0.427	1.028	3.146
1Y4H	A:B	207	15	0.072	1.400	0.443	1.228	2.825
1Y96	A:C	156	7	0.045	1.000	0.369	0.912	3.469
1YAD	A:D	129	4	0.031	1.250	0.408	0.971	3.002
1YC2	A:D	267	7	0.026	1.429	0.377	1.311	3.685
1YDG	C:F	104	4	0.038	1.000	0.440	0.963	2.940
1YFN	A:C	115	8	0.070	1.375	0.421	1.216	3.553
1YNH	B:D	147	8	0.054	1.125	0.460	1.207	3.348
1YO6	A:C	194	6	0.031	1.500	0.410	1.335	4.316
1YQF	A:F	135	6	0.044	1.000	0.403	0.944	3.003
1YU6	A:B	127	8	0.063	1.250	0.387	1.194	2.857
1YUP	D:E	189	10	0.053	1.400	0.341	1.050	3.657
1Z0K	B:C	106	13	0.123	1.538	0.473	1.132	3.284
1Z2I	B:C	298	12	0.040	1.000	0.353	0.968	4.043
1ZHQ	D:G	145	16	0.110	1.250	0.403	1.133	3.617
1ZKE	A:B	116	8	0.069	1.375	0.398	1.199	3.216
1ZOQ	A:D	116	9	0.078	1.000	0.402	0.959	3.417
1ZVP	A:B	180	11	0.061	1.455	0.408	1.064	3.364
1ZW0	C:G	124	5	0.040	1.800	0.361	1.395	2.934
2A1S	A:C	300	2	0.007	1.500	0.406	1.679	3.876
2A7K	F:I	147	5	0.034	1.400	0.437	1.715	3.424
2AAG	A:D	129	12	0.093	1.333	0.376	1.062	2.889
2ABJ	A:G	180	13	0.072	1.308	0.413	1.245	3.209
2ASH	A:C	173	17	0.098	1.176	0.404	0.979	3.152
2AYE	A:C	119	3	0.025	1.000	0.388	0.915	2.763
2B30	B:C	195	1	0.005	1.000	0.340	0.975	3.364
2BEX	A:B	185	18	0.097	1.111	0.443	1.195	3.775
2BHM	B:E	177	1	0.006	1.000	0.409	1.017	3.120
2BKK	B:C	111	8	0.072	1.250	0.379	1.250	3.086
2BS9	C:E	113	1	0.009	2.000	0.393	2.194	3.442
2BYK	A:C	149	7	0.047	1.286	0.366	1.168	4.029
2BYK	A:D	108	3	0.028	1.667	0.343	1.259	3.134
2BYK	B:D	133	3	0.023	1.000	0.331	1.039	2.892
2BYS	E:I	201	18	0.090	1.278	0.377	1.023	3.551
2C21	B:C	256	37	0.145	1.595	0.397	0.986	3.626
2C6Q	D:H	198	19	0.096	1.158	0.385	0.984	3.495
2CH5	B:D	167	12	0.072	1.333	0.419	1.265	3.756
2CJR	B:C	172	12	0.070	1.417	0.388	1.149	3.705
2CW6	C:E	197	6	0.030	1.167	0.335	1.256	4.508
2D04	B:F	122	2	0.016	1.000	0.450	0.847	2.675
2D0J	A:D	162	14	0.086	1.357	0.453	1.208	3.104

2D4C	A:D	134	1	0.007	1.000	0.436	0.971	3.286
2D5W	A:B	351	31	0.088	1.452	0.394	1.103	4.130
2D7E	A:C	194	2	0.010	1.500	0.370	1.617	3.416
2DFU	B:D	147	6	0.041	1.167	0.404	0.997	3.221
2DS2	B:D	116	3	0.026	1.667	0.372	1.534	2.383
2DSB	B:C	220	4	0.018	1.000	0.333	1.063	2.800
2DVM	A:C	371	27	0.073	1.556	0.422	0.963	3.505
2DWX	A:B	329	9	0.027	1.333	0.338	1.037	4.958
2DWZ	A:C	198	5	0.025	1.200	0.326	1.170	2.861
2DZN	B:D	104	1	0.010	1.000	0.320	1.010	2.800
2E11	C:D	189	8	0.042	1.125	0.403	0.847	2.956
2E21	B:C	195	6	0.031	1.167	0.407	1.110	3.103
2E7L	B:E	110	1	0.009	1.000	0.477	1.294	2.651
2ED6	E:I	107	6	0.056	1.000	0.446	1.244	2.844
2EFC	A:C	182	9	0.049	1.000	0.382	0.805	3.215
2EJW	B:E	376	21	0.056	1.143	0.366	0.972	3.398
2EK6	A:D	129	4	0.031	1.000	0.336	0.992	2.706
2EKY	B:F	108	6	0.056	1.000	0.382	0.783	3.423
2EQ5	B:C	205	9	0.044	1.556	0.398	1.281	2.985
2EY4	A:B	188	9	0.048	1.111	0.408	0.976	3.564
2F2F	B:E	168	6	0.036	1.333	0.426	1.310	2.860
2FDB	P:R	326	15	0.046	1.333	0.421	1.138	3.434
2FEN	H:J	218	3	0.014	1.000	0.442	1.389	4.003
2FIP	A:C	138	10	0.072	1.200	0.406	1.134	3.071
2FU5	A:B	224	15	0.067	1.200	0.421	0.908	3.631
2FV2	A:C	128	8	0.063	1.000	0.450	0.895	2.900
2G56	A:B	422	14	0.033	1.143	0.377	1.037	4.629
2GA0	B:F	115	4	0.035	1.500	0.405	1.487	3.334
2GA6	I:M	147	5	0.034	1.600	0.415	1.285	2.905
2GD0	A:C	152	14	0.092	1.357	0.413	1.109	3.176
2GEY	A:D	198	10	0.051	1.000	0.394	0.832	3.260
2GEZ	C:E	154	1	0.006	1.000	0.392	0.981	2.825
2GIN	C:F	150	8	0.053	1.000	0.303	0.974	3.793
2GT2	A:D	173	16	0.092	1.813	0.401	1.201	4.340
2GUZ	A:C	141	6	0.043	1.500	0.400	1.429	2.985
2GUZ	B:F	303	31	0.102	1.581	0.382	0.932	3.385
2GUZ	B:M	138	20	0.145	1.250	0.398	1.058	3.508
2GVG	C:E	138	3	0.022	1.333	0.400	1.227	3.054
2GWF	A:E	125	2	0.016	1.000	0.407	1.582	3.114
2GWF	B:F	104	4	0.038	1.000	0.530	0.972	2.661
2GWF	C:F	131	4	0.031	1.250	0.386	1.129	3.233
2GZ4	A:D	111	15	0.135	1.267	0.438	1.098	3.248
2HDN	D:H	125	1	0.008	1.000	0.403	1.453	3.445
2HLN	A:C	155	15	0.097	1.333	0.393	1.123	3.705
2HPA	A:D	158	3	0.019	1.000	0.387	1.254	3.399
2HQH	A:C	122	5	0.041	1.000	0.419	1.008	2.730
2HZM	A:D	289	21	0.073	1.524	0.384	0.927	3.930
2HZM	D:F	230	9	0.039	1.222	0.394	0.959	3.557
2I00	B:D	158	2	0.013	1.500	0.359	1.500	2.889
2I1A	A:D	167	11	0.066	1.455	0.410	1.349	3.638
2I25	N:M	102	7	0.069	1.143	0.453	0.980	2.965
2I2W	C:D	300	16	0.053	1.375	0.408	1.066	3.597
2I39	A:D	234	17	0.073	1.765	0.433	1.290	3.212
2I79	A:B	143	6	0.042	1.667	0.387	1.324	2.775
2I7P	A:B	232	3	0.013	1.000	0.428	1.373	3.144
2IA5	B:G	154	8	0.052	1.250	0.397	1.153	3.228

2IBG	B:C	268	10	0.037	1.400	0.407	1.369	4.182
2IDE	E:H	133	12	0.090	1.917	0.347	1.301	3.002
2IDO	A:C	247	16	0.065	1.375	0.398	1.071	4.247
2IQ7	A:C	218	15	0.069	1.333	0.502	1.167	4.033
2ISK	A:E	174	12	0.069	1.667	0.395	1.255	3.361
2IV3	A:C	110	3	0.027	1.000	0.421	1.078	2.649
2J0Y	B:F	133	5	0.038	1.200	0.383	0.967	3.441
2J1K	K:X	116	3	0.026	1.000	0.398	0.859	2.860
2J4E	B:F	135	2	0.015	1.000	0.429	1.216	2.841
2J5G	B:L	175	19	0.109	1.158	0.410	1.050	3.739
2J8X	A:C	269	21	0.078	1.143	0.403	0.979	4.575
2JBM	F:I	139	10	0.072	1.400	0.364	1.201	3.111
2JH3	A:D	420	32	0.076	1.438	0.410	1.230	5.273
2JJB	A:B	143	9	0.063	1.222	0.418	1.106	3.502
2JKV	C:F	165	9	0.055	1.222	0.429	1.050	2.843
2NOX	D:F	175	11	0.063	1.818	0.457	1.174	2.778
2NWI	A:C	140	8	0.057	1.125	0.439	1.003	3.501
2NX4	A:D	315	26	0.083	1.385	0.412	0.894	3.600
2NXI	A:G	150	13	0.087	1.154	0.350	0.883	3.585
2NZO	B:D	154	10	0.065	2.000	0.389	1.351	3.874
2O70	C:F	143	8	0.056	1.125	0.326	1.141	3.320
2O9A	A:B	311	25	0.080	1.480	0.374	1.007	4.370
2ODE	B:C	148	15	0.101	1.333	0.398	1.102	3.588
2ODF	C:D	155	10	0.065	1.000	0.428	1.123	2.982
2OIF	B:F	153	12	0.078	1.250	0.376	1.034	3.048
2OJ5	C:E	111	14	0.126	1.286	0.412	0.971	3.362
2OPO	B:C	162	12	0.074	1.167	0.413	1.062	3.223
2OQ2	A:D	196	7	0.036	1.000	0.392	1.032	3.997
2OVI	A:B	182	9	0.049	1.778	0.364	1.451	3.002
2OXG	B:F	226	24	0.106	1.417	0.411	0.897	3.153
2P0W	A:B	155	7	0.045	1.000	0.372	0.975	3.515
2P1N	B:E	185	3	0.016	1.000	0.363	0.822	2.961
2PKD	A:C	242	16	0.066	1.250	0.381	1.065	3.501
2PQR	B:C	103	12	0.117	1.667	0.418	1.253	2.612
2Q08	C:F	152	8	0.053	1.000	0.451	1.109	3.091
2Q5Y	A:C	165	5	0.030	1.000	0.381	1.025	3.302
2Q8R	F:H	142	9	0.063	1.333	0.421	1.076	2.606
2Q8T	B:D	161	15	0.093	1.600	0.349	1.244	3.537
2Q9Q	A:G	158	6	0.038	1.167	0.421	1.145	3.501
2QKE	A:C	112	2	0.018	1.500	0.327	1.344	3.144
2QKP	A:C	153	6	0.039	1.167	0.442	1.008	3.274
2QLP	A:F	149	2	0.013	1.000	0.327	0.931	3.262
2QMS	A:D	150	6	0.040	1.167	0.451	1.108	3.202
2QQ4	A:C	120	7	0.058	1.143	0.434	1.016	2.819
2QTS	A:E	126	7	0.056	1.000	0.429	1.326	3.275
2QUN	B:C	260	24	0.092	1.333	0.407	1.080	3.233
2R6U	C:D	175	24	0.137	1.708	0.417	1.103	3.774
2R7A	C:D	198	9	0.045	1.333	0.392	1.307	3.956
2R9G	C:M	173	6	0.035	1.500	0.407	1.324	3.167
2RAG	B:C	203	14	0.069	1.143	0.376	0.991	3.283
2RB3	A:B	169	12	0.071	1.167	0.363	0.952	3.509
2RB9	B:D	279	29	0.104	1.483	0.396	1.146	4.063
2RC3	A:B	247	21	0.085	1.238	0.385	0.959	3.395
2RDZ	A:D	206	26	0.126	1.423	0.411	1.058	3.548
2RKL	B:D	118	7	0.059	1.429	0.351	1.297	3.009
2UUV	A:C	210	21	0.100	1.429	0.392	1.128	3.612

2V3S	A:B	149	13	0.087	1.154	0.382	1.023	3.778
2V4M	A:C	141	3	0.021	2.000	0.413	1.831	3.585
2V57	A:D	108	5	0.046	1.200	0.417	1.271	3.064
2V5Q	C:D	125	3	0.024	1.000	0.402	0.984	3.288
2V74	B:F	185	5	0.027	1.000	0.383	0.959	3.369
2V8W	A:E	175	13	0.074	1.154	0.383	0.995	3.424
2VAT	A:B	320	24	0.075	1.375	0.399	0.995	3.602
2VG2	A:B	233	13	0.056	1.077	0.391	0.804	4.072
2VLG	A:B	146	6	0.041	1.000	0.350	0.816	2.911
2VNV	B:C	142	16	0.113	1.625	0.405	1.304	3.647
2VPF	B:E	165	13	0.079	1.538	0.401	1.308	3.325
2VRF	B:D	117	2	0.017	1.000	0.409	1.009	2.855
2VUT	A:G	278	19	0.068	1.789	0.398	1.103	3.261
2VUT	A:K	122	6	0.049	1.500	0.362	1.245	3.395
2VV6	B:D	145	15	0.103	1.067	0.415	0.899	3.291
2VVP	B:D	220	20	0.091	1.000	0.420	0.982	3.928
2VZD	A:B	156	2	0.013	1.500	0.266	1.322	3.448
2W01	B:C	112	3	0.027	1.333	0.413	1.185	2.776
2W2D	A:C	185	11	0.059	1.364	0.397	1.225	3.489
2W2D	B:C	125	9	0.072	1.667	0.397	1.319	3.156
2W2D	B:D	164	4	0.024	1.500	0.388	1.952	3.282
2W2U	A:B	128	6	0.047	1.000	0.369	0.883	3.002
2WAY	A:C	187	5	0.027	1.000	0.385	0.850	3.151
2WEU	B:C	361	24	0.066	1.875	0.421	1.209	3.811
2WGS	D:H	749	20	0.027	2.050	0.353	1.078	6.198
2WOJ	A:D	112	4	0.036	1.000	0.463	1.047	3.006
2WOW	B:C	177	8	0.045	1.000	0.444	1.006	3.416
2WPV	A:C	371	41	0.111	1.439	0.412	1.009	3.473
2WPV	A:D	218	21	0.096	1.714	0.355	0.994	2.942
2WTM	B:C	121	6	0.050	1.000	0.426	0.917	2.715
2WTX	A:B	384	23	0.060	1.391	0.368	1.039	4.035
2WYA	A:B	280	28	0.100	1.393	0.417	1.108	3.881
2WYF	D:G	158	7	0.044	1.143	0.430	0.971	3.070
2X2S	A:C	103	12	0.117	1.250	0.374	1.170	3.134
2X36	C:E	108	1	0.009	1.000	0.411	0.973	2.715
2XCM	A:D	240	7	0.029	1.429	0.395	1.275	3.593
2XE5	A:D	133	8	0.060	1.250	0.424	1.299	3.073
2XEC	A:B	266	2	0.008	2.500	0.432	1.591	2.493
2XEW	D:H	173	5	0.029	1.000	0.345	0.783	3.246
2XLA	B:C	110	5	0.045	1.000	0.352	1.038	3.720
2YW3	C:D	141	2	0.014	1.000	0.410	0.959	2.676
2YWB	A:C	757	34	0.045	1.412	0.391	1.148	4.682
2YYT	A:B	103	2	0.019	1.000	0.426	0.981	2.766
2Z0J	E:G	183	13	0.071	1.385	0.412	1.136	3.420
2Z0P	A:D	143	3	0.021	1.000	0.379	1.036	3.167
2Z3R	E:H	153	8	0.052	1.375	0.372	1.216	2.934
2Z3R	F:H	110	5	0.045	1.200	0.429	1.148	3.254
2Z5I	E:G	116	7	0.060	1.000	0.440	0.928	2.529
2Z5S	N:O	129	4	0.031	1.500	0.376	1.524	2.946
2Z8J	B:C	135	3	0.022	1.000	0.424	0.971	3.841
2ZCI	C:D	257	4	0.016	1.000	0.415	1.008	2.779
2ZF3	A:F	167	6	0.036	1.500	0.354	1.606	3.570
2ZNW	A:Z	135	4	0.030	1.500	0.458	1.235	2.716
2ZON	A:G	186	8	0.043	1.125	0.360	0.868	3.199
2ZUT	A:C	258	28	0.109	1.143	0.383	1.072	4.138
2ZV3	B:F	302	21	0.070	1.286	0.388	0.916	3.258

2ZZX	A:C	172	16	0.093	1.438	0.359	1.177	3.342
3A18	A:D	310	23	0.074	1.565	0.453	1.301	3.421
3A1P	A:C	230	18	0.078	1.111	0.458	1.052	4.033
3A2A	A:D	148	3	0.020	1.000	0.366	1.021	2.697
3A5P	A:C	160	20	0.125	1.500	0.379	1.101	3.449
3A8Y	C:D	366	7	0.019	1.571	0.365	1.445	3.822
3AAA	A:C	171	11	0.064	1.727	0.381	0.998	3.702
3AAA	B:C	186	14	0.075	1.429	0.413	1.059	3.526
3AB4	K:M	188	3	0.016	1.000	0.395	0.984	3.636
3AL4	A:F	136	3	0.022	1.000	0.391	0.834	2.805
3AL4	C:I	147	1	0.007	1.000	0.473	1.441	3.328
3AL4	H:J	361	4	0.011	1.250	0.350	1.014	3.084
3B4V	D:G	206	4	0.019	1.250	0.421	1.355	3.402
3B5M	A:C	114	14	0.123	1.214	0.430	1.099	3.268
3B9J	C:K	888	37	0.042	1.486	0.395	1.025	8.028
3BAT	B:D	121	6	0.050	1.000	0.391	1.163	2.626
3BFW	A:C	119	10	0.084	1.300	0.413	1.060	3.026
3BNY	A:C	239	15	0.063	1.333	0.446	1.374	4.524
3BPJ	B:C	125	3	0.024	1.000	0.402	0.880	3.506
3BU8	A:B	173	11	0.064	1.545	0.438	1.494	3.559
3BX1	A:D	257	21	0.082	1.619	0.403	0.986	3.860
3BX1	D:C	280	23	0.082	1.391	0.381	0.994	4.486
3C0V	C:D	216	16	0.074	1.125	0.370	0.910	3.328
3C2X	A:C	126	12	0.095	1.500	0.395	1.260	3.019
3C66	A:B	141	3	0.021	1.000	0.355	1.007	3.339
3C8D	B:D	132	10	0.076	1.000	0.402	1.091	3.383
3C9A	A:B	186	15	0.081	1.600	0.357	1.235	4.012
3C9A	B:C	144	11	0.076	1.455	0.353	1.080	3.081
3CB0	A:B	140	10	0.071	1.300	0.385	1.034	3.583
3CFI	A:J	173	3	0.017	1.000	0.359	0.972	3.124
3CIS	A:C	189	5	0.026	1.000	0.370	0.883	2.937
3CJS	A:C	110	3	0.027	1.333	0.374	1.346	2.729
3CMM	A:C	467	4	0.009	1.000	0.387	1.229	3.691
3CPH	G:H	131	1	0.008	1.000	0.400	0.992	2.993
3D4I	A:D	307	10	0.033	1.200	0.384	1.023	3.990
3DAB	C:G	136	10	0.074	1.000	0.421	0.800	3.380
3DAQ	B:C	258	28	0.109	1.286	0.404	0.993	3.635
3DCG	A:C	332	20	0.060	1.450	0.391	1.075	3.962
3DGA	A:D	214	3	0.014	2.000	0.327	1.461	3.429
3DGV	B:C	228	9	0.039	1.556	0.370	1.119	2.832
3DHO	B:D	119	6	0.050	1.000	0.398	1.167	2.756
3DKU	E:G	128	3	0.023	1.333	0.440	1.130	2.620
3DQG	B:D	236	20	0.085	1.300	0.435	1.096	3.607
3DTD	I:J	138	5	0.036	1.000	0.398	0.939	3.488
3DUH	A:B	285	10	0.035	1.000	0.444	1.063	3.472
3DVT	B:C	146	11	0.075	1.364	0.467	1.070	3.741
3DX9	A:D	112	1	0.009	1.000	0.432	1.318	2.695
3DXC	A:C	199	4	0.020	1.500	0.426	1.144	2.773
3E05	B:G	152	3	0.020	1.000	0.376	1.226	3.357
3E1E	B:D	175	8	0.046	1.625	0.419	1.429	3.333
3E2P	B:J	149	5	0.034	1.200	0.403	0.956	3.245
3E7D	A:C	152	9	0.059	1.000	0.448	1.126	3.677
3EBN	A:D	135	1	0.007	1.000	0.425	1.007	2.443
3ED4	A:D	212	10	0.047	1.300	0.376	1.120	4.342
3EEY	B:C	224	9	0.040	1.222	0.386	1.108	4.059
3EFE	A:B	165	13	0.079	1.462	0.355	1.171	3.262

3EGG	A:B	178	16	0.090	1.375	0.414	1.088	3.512
3EJB	B:D	298	15	0.050	1.267	0.406	1.302	4.034
3ERY	A:B	349	16	0.046	1.563	0.417	1.035	3.789
3EW1	D:F	557	34	0.061	1.206	0.388	0.925	4.147
3EWZ	B:C	209	21	0.100	1.476	0.330	1.234	3.543
3EZQ	I:M	127	3	0.024	1.667	0.395	1.764	2.115
3F0Y	B:G	182	14	0.077	1.357	0.435	1.029	3.692
3F2O	A:B	133	15	0.113	1.600	0.373	1.176	3.505
3FA4	F:K	125	5	0.040	1.400	0.475	1.207	3.274
3FAV	A:C	109	10	0.092	1.100	0.394	1.061	3.205
3FGN	A:C	121	5	0.041	1.000	0.388	0.992	2.880
3FHL	B:C	166	4	0.024	1.250	0.389	1.281	3.633
3FJG	A:D	126	11	0.087	1.182	0.400	0.942	2.814
3FNM	B:D	103	4	0.039	2.000	0.313	1.609	2.902
3FSH	A:B	162	1	0.006	1.000	0.391	1.006	3.015
3FXD	B:D	339	9	0.027	1.556	0.358	1.174	3.289
3G03	A:C	150	12	0.080	1.417	0.326	1.101	3.223
3G17	A:B	301	19	0.063	1.316	0.426	1.062	3.093
3G2S	A:B	317	38	0.120	1.605	0.401	0.996	3.788
3G3A	C:F	143	10	0.070	1.200	0.376	1.028	3.453
3G6N	A:B	133	1	0.008	1.000	0.386	1.167	2.647
3G7G	A:F	105	7	0.067	2.286	0.459	1.472	2.727
3G8Q	C:D	144	1	0.007	1.000	0.427	1.143	3.110
3G9V	A:C	225	2	0.009	1.000	0.444	1.061	3.875
3GFU	C:A	196	10	0.051	1.700	0.371	1.216	3.308
3GJ4	A:C	192	16	0.083	1.500	0.335	1.190	3.976
3GJS	A:C	101	6	0.059	1.333	0.484	1.448	2.731
3GJS	A:D	138	11	0.080	1.182	0.433	1.032	2.686
3GJS	B:D	434	27	0.062	1.519	0.339	0.860	3.363
3GJX	A:D	135	2	0.015	1.000	0.414	1.286	2.935
3GMW	A:C	231	6	0.026	1.000	0.347	0.885	2.772
3GN8	A:B	120	1	0.008	1.000	0.370	1.081	2.702
3GOF	A:B	177	11	0.062	1.000	0.422	1.006	3.284
3GQ1	A:B	121	5	0.041	1.400	0.414	1.219	2.789
3GWC	B:F	171	12	0.070	1.250	0.377	0.946	2.996
3GZ8	B:C	192	8	0.042	1.250	0.375	1.053	2.879
3GZF	A:D	112	1	0.009	1.000	0.351	1.018	2.994
3H16	A:C	187	5	0.027	1.600	0.423	1.268	3.169
3H3B	A:B	170	15	0.088	1.133	0.310	0.983	3.794
3H3B	A:D	155	9	0.058	1.222	0.438	1.128	3.017
3H8D	B:C	177	19	0.107	1.316	0.411	1.008	3.724
3H8D	E:H	102	11	0.108	1.364	0.462	1.312	2.689
3H90	A:B	135	2	0.015	1.000	0.398	1.000	3.294
3HA4	A:F	142	7	0.049	1.429	0.385	1.380	3.077
3HE4	A:H	111	1	0.009	1.000	0.336	1.261	2.522
3HEI	A:N	155	21	0.135	1.524	0.366	1.074	3.379
3HEI	E:K	250	18	0.072	1.333	0.366	1.134	3.862
3HEI	L:P	228	28	0.123	1.393	0.420	1.062	3.782
3HG0	A:D	123	4	0.033	1.000	0.387	0.848	2.893
3HHD	A:B	158	6	0.038	1.000	0.388	1.534	3.372
3HM7	A:E	229	10	0.044	2.300	0.370	1.606	3.021
3HP3	D:F	281	4	0.014	1.000	0.397	0.953	3.585
3HSR	B:D	104	4	0.038	1.000	0.450	1.000	3.112
3HTA	A:C	165	7	0.042	1.143	0.392	0.998	3.033
3HTU	B:D	207	2	0.010	1.000	0.346	1.107	2.919
3HWK	B:D	318	28	0.088	1.357	0.410	1.023	4.023

3I26	A:D	200	16	0.080	1.125	0.440	1.047	3.471
3I2B	D:I	281	10	0.036	1.200	0.450	0.955	3.632
3ICI	A:B	106	7	0.066	1.000	0.455	1.178	3.417
3IJ6	A:C	101	3	0.030	1.000	0.449	1.074	3.168
3IML	A:C	139	2	0.014	1.000	0.394	0.869	2.666
3IPR	A:F	130	2	0.015	1.500	0.430	1.291	2.918
3IX0	B:D	110	6	0.055	1.500	0.394	1.398	2.908
3IX4	C:G	139	7	0.050	1.143	0.402	1.012	2.823
3JSX	A:D	139	6	0.043	1.167	0.383	1.229	3.519
3K2B	A:G	102	2	0.020	1.000	0.370	1.020	2.955
3K2M	C:D	196	6	0.031	1.667	0.384	1.541	2.639
3K2Y	B:C	119	4	0.034	1.500	0.417	1.352	2.870
3K4W	C:E	230	14	0.061	1.214	0.407	0.922	3.197
3K9M	C:B	143	2	0.014	1.500	0.397	1.543	3.000
3K9X	A:D	255	19	0.075	1.526	0.377	1.236	3.734
3KAW	A:C	123	11	0.089	1.455	0.402	1.140	2.907
3KD2	C:D	294	41	0.139	1.854	0.415	1.126	3.789
3KF8	B:C	140	3	0.021	1.000	0.321	0.875	3.791
3KIK	A:D	134	5	0.037	1.000	0.341	0.832	3.583
3KO5	B:C	145	10	0.069	1.000	0.452	0.986	3.210
3KP1	A:F	209	28	0.134	1.321	0.414	0.952	3.727
3KS0	L:A	104	1	0.010	1.000	0.340	1.195	3.102
3KSC	B:E	357	12	0.034	1.750	0.452	1.161	3.449
3KT3	A:C	110	4	0.036	1.250	0.434	1.136	2.745
3KTM	A:E	385	16	0.042	1.625	0.371	1.515	5.187
3KUS	A:B	143	9	0.063	1.222	0.351	1.079	2.942
3KW1	D:H	160	11	0.069	1.545	0.510	0.951	3.687
3KWC	A:E	127	9	0.071	1.000	0.415	0.992	3.358
3KWM	A:C	260	13	0.050	1.462	0.413	1.191	4.177
3L2E	A:D	234	2	0.009	1.000	0.345	1.322	4.770
3L43	B:D	120	3	0.025	1.000	0.453	1.017	3.437
3L49	B:D	102	1	0.010	1.000	0.396	1.146	3.204
3L8R	C:D	133	2	0.015	1.500	0.389	1.571	2.695
3LB5	B:D	269	22	0.082	1.591	0.429	1.135	3.576
3LCP	A:B	144	4	0.028	1.250	0.493	1.364	3.049
3LG6	A:C	181	17	0.094	1.000	0.360	0.801	3.858
3LPE	C:E	166	6	0.036	1.333	0.388	1.258	3.845
3LT7	C:D	250	13	0.052	1.154	0.409	1.131	2.859
3LW8	C:G	127	11	0.087	1.000	0.440	1.033	3.549
3LXT	B:D	184	9	0.049	1.000	0.429	1.034	3.042
3LYF	B:C	123	6	0.049	1.000	0.436	0.885	3.658
3M3I	C:E	113	1	0.009	1.000	0.438	1.108	2.480
3M4W	B:C	154	6	0.039	1.333	0.439	1.141	4.033
3M5R	A:G	355	15	0.042	1.267	0.379	1.078	5.413
3MAV	B:C	107	9	0.084	1.111	0.367	0.991	2.924
3MCE	A:C	121	2	0.017	1.000	0.277	0.877	3.211
3MDY	A:D	125	6	0.048	1.333	0.412	1.048	3.178
3MHF	A:C	317	49	0.155	1.837	0.403	1.068	3.820
3MJH	B:C	110	3	0.027	1.333	0.383	1.192	2.692
3MO2	B:C	114	4	0.035	1.500	0.482	1.336	3.398
3MQH	C:F	106	7	0.066	1.000	0.414	1.082	2.691
3N1F	B:D	152	21	0.138	1.667	0.443	1.206	3.416
3N1G	B:A	150	18	0.120	1.278	0.409	0.954	3.067
3N25	C:F	135	3	0.022	1.000	0.386	1.047	3.210
3NBS	A:D	128	9	0.070	1.556	0.328	1.171	3.105
3NGB	H:D	198	5	0.025	1.000	0.368	0.943	3.269

3NMN	B:D	155	2	0.013	1.000	0.379	1.047	3.215
3NRJ	A:E	129	8	0.062	1.000	0.388	0.970	3.315
5PGM	A:G	166	12	0.072	1.250	0.383	1.482	3.547
